# Meta-analysis of natural killer cell cytotoxicity in myalgic encephalomyelitis/chronic fatigue syndrome

**DOI:** 10.3389/fimmu.2024.1440643

**Published:** 2024-10-17

**Authors:** James N. Baraniuk, Natalie Eaton-Fitch, Sonya Marshall-Gradisnik

**Affiliations:** ^1^ Department of Medicine, Georgetown University, Washington, DC, United States; ^2^ National Centre for Neuroimmunology and Emerging Diseases, Griffith University, Gold Coast, QLD, Australia

**Keywords:** meta-analysis, natural killer cells, cytotoxicity, myalgic encephalomyelitis/chronic fatigue syndrome, ME/CFS

## Abstract

Reduced natural killer (NK) cell cytotoxicity is the most consistent immune finding in myalgic encephalomyelitis/chronic fatigue syndrome (ME/CFS). Meta-analysis of the published literature determined the effect size of the decrement in ME/CFS. Databases were screened for papers comparing NK cell cytotoxicity in ME/CFS and healthy controls. A total of 28 papers and 55 effector:target cell ratio (E:T) data points were collected. Cytotoxicity in ME/CFS was significantly reduced to about half of healthy control levels, with an overall Hedges’ g of 0.96 (0.75–1.18). Heterogeneity was high but was explained by the range of E:T ratios, different methods, and potential outliers. The outcomes confirm reproducible NK cell dysfunction in ME/CFS and will guide studies using the NK cell model system for pathomechanistic investigations.

**Systematic review registration:**

https://www.crd.york.ac.uk/prospero/, identifier CRD42024542140.

## Introduction

Myalgic encephalomyelitis/chronic fatigue syndrome (ME/CFS) is a chronic disease with disability, fatigue, post-exertional malaise (PEM), cognitive lapses, non-refreshing sleep, interoceptive distress, pain, and orthostatic complaints (1–5). PEM is the key manifestation. Physical, cognitive, or emotional exertion at greater than usual levels leads to symptom relapse that may be of immediate onset or delayed by hours and that forces patients to remain in bed or house bound until recovery. Patients often recall a severe flu-like illness that never resolved. There have been epidemic outbreaks, but most cases are sporadic. The symptom profile mirrors long coronavirus disease 2019 (COVID), the persistent fatiguing illness that does not abate after acute severe acute respiratory syndrome coronavirus 2 (SARS-CoV-2) infection (6) and that was predicted to be a precursor to ME/CFS (7). Multiple lines of evidence suggest that ME/CFS and long COVID are parallel symptom complexes that share brain, metabolic, and immune pathologies (8). There are no diagnostic tests or approved therapies for either disorder.

Natural killer (NK) cell dysfunction has been observed in ME/CFS since 1987 (9) and contributes to the hypothesis of immune dysfunction in ME/CFS. In contrast, other measures such as aberrations in pro- and anti-inflammatory cytokines, lymphocyte populations, autoantibodies, metabolomics, and functional assays have not been shown to be reproducible (10). NK cell dysfunction is supported by extensive research into changes in NK cell phenotype profiles, surface regulatory receptors, reduced store-operated calcium levels, significantly reduced lytic protein production, and release. Cytotoxicity assesses all steps of NK cell activation from receptor-ligand binding, kinase pathway and calcium mobilization, microtubule and granule polarization, degranulation of dense and light granules with release of their contents, upregulation of markers of degranulation, and subsequent apoptosis with loss of membrane integrity of the target cells.

The K562 human erythroleukemia tumor cell line is the standard target cell to assess NK cell function *in vitro*. K562 cells do not express major histocompatibility complex class I (MHC-I) human leukocyte antigens (HLA)-A, HLA-B, or HLA-C that would normally be recognized by NK cell killer inhibitory receptors (KIR) and prevent cytotoxicity (11).

There are two major methods to measure NK cell cytotoxicity. The traditional “gold standard” ^51^Chromium (^51^Cr) release assay involves loading ^51^Cr into K562 cells, which are then incubated with whole blood, peripheral blood mononuclear cells (PBMCs), or isolated NK cells at effector to target cell (E:T) ratios of 50:1 to 6.25:1 for 4 h at 37°C in 5% carbon dioxide (12–15). NK cell cytotoxicity is quantitatively measured by the release of ^51^Cr into the supernatant and calculation of percentage (%) killing at each E:T ratio. A non-radioactive alternative method is fluorescent cytometry to detect NK-cell-induced apoptosis of K562 cells with expression of cell surface phosphatidylserine by binding fluorescently tagged annexin V (16). Unfortunately, both methods are labor intensive, require special safety measures, and are not approved as diagnostic clinical tests (17) for ME/CFS.

This meta-analysis was performed to quantify the effect size (Hedges’ g) for the difference in % cytotoxicity of NK cells between ME/CFS and healthy controls (HC). The literature was reviewed to improve understanding of the details of these tests. An innovation was to extract data and analyze the effects of individual E:T ratios and the extrapolated outcomes.

## Results

### Overview

**Figure 1:**
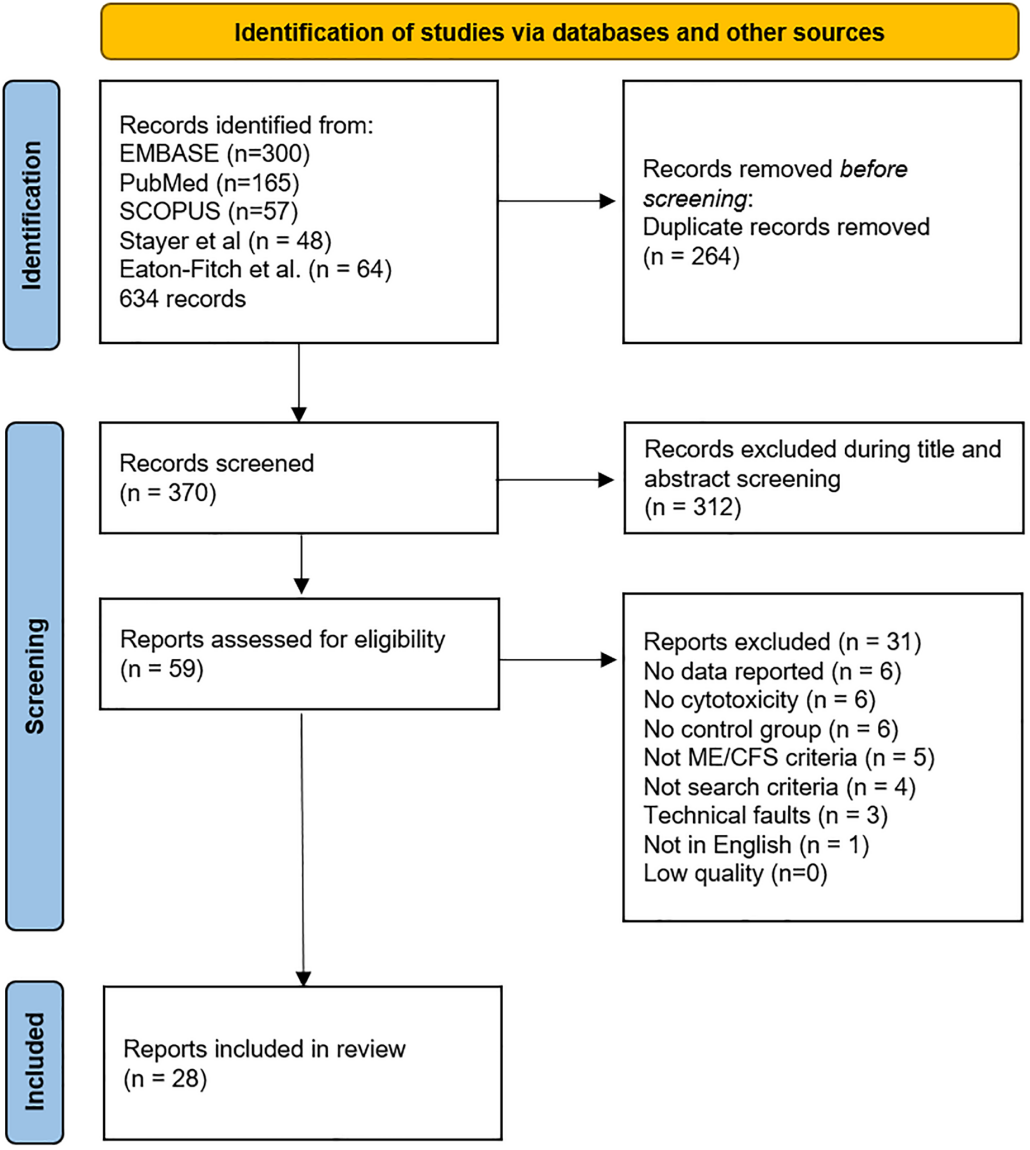
PRISMA flow diagram demonstrating searches of databases and other sources completed by JNB.

The review process selected 28 manuscripts for the final meta-analysis (**Figure 1** and **Table 1**). Title and abstract screening was completed for 579 records obtained from literature reviews by Strayer et al. (19), Eaton-Fitch et al. (10), and an additional review of the literature to include more recent publications by JNB. Strayer et al. cited referenced 48 references with 27 papers related to NK cell cytotoxicity. Eaton-Fitch et al. (10) identified a total of 523 papers from Medline (EBSCOhost) (n=111), Embase (n=159), PubMed (n=73), and Scopus (n=180) databases. Their review cited 64 manuscripts, but their analysis was limited to 17 NK cell function papers.

**Table 1:** Data for each E:T ratio. Hedges’ g between ME/CFS and HC was determined for each E:T ratio.

Row	Cell	Reference	E:T	ME	HC	ME/HC	Hedges’ g (95% CI)
1	WB ^51^Cr	(12) Klimas 1990	1	9 ± 9.5 (13)	25 ± 12.6 (69)	0.360	1.301 (0.684–1.942)
2	WB ^51^Cr	(13) Fletcher 2002	1	21 ± 10.5 (29)	36 ± 19 (29)	0.583	0.964 (0.428–1.526)
3	WB ^51^Cr	(14) Maher 2005	1	18 ± 20 (30)	50 ± 40 (19)	0.360	1.074 (0.471–1.712)
4	WB ^51^Cr	(15) Fletcher 2010	1	12 ± 14.6 (176)	28 ± 17.8 (230)	0.429	0.968 (0.763–1.178)
5	WB ^51^Cr	(69) Masuda 1994	10	5.7 ± 2.8 (10)	22.5 ± 11.9 (21)	0.253	1.635 (0.804–2.554)
6	WB ^51^Cr	(69) Masuda 1994	20	8.8 ± 4.4 (10)	31.5 ± 17.4 (21)	0.279	1.508 (0.689–2.409)
7	WB ^51^Cr	(69) Masuda 1994	10	13.6 ± 9.8 (24)	22.5 ± 11.9 (21)	0.604	0.808 (0.206–1.438)
8	WB ^51^Cr	(69) Masuda 1994	20	18.7 ± 12.2 (24)	31.5 ± 17.4 (21)	0.594	0.846 (0.242–1.480)
9	WB ^51^Cr	(70) Masuda 2002	10	7.2 ± 4.1 (16)	26.7 ± 9.7 (20)	0.270	2.462 (1.628–3.407)
10	WB ^51^Cr	(70) Masuda 2002	20	12.3 ± 3.8 (16)	35.9 ± 12.5 (20)	0.343	2.384 (1.561–3.316)
11	WB ^51^Cr	(70) Masuda 2002	10	4.4 ± 2.3 (20)	26.7 ± 9.7 (20)	0.165	3.101 (2.222–4.105)
12	WB ^51^Cr	(70) Masuda 2002	20	6.5 ± 2.9 (20)	35.9 ± 12.5 (20)	0.181	3.176 (2.286–4.194)
13	PBMC ^51^Cr	(9) Caligiuri 1987	30	26 ± 19.2 (41)	46 ± 15 (25)	0.557	1.114 (0.590–1.664)
14	PBMC ^51^Cr	(9) Caligiuri 1987	60	37 ± 16 (8)	58 ± 14 (6)	0.638	1.293 (0.158–2.605)
15	PBMC ^51^Cr	(71) Mawle 1997	50	32.5 ± 16.9 (26)	29.4 ± 17.6 (50)	1.105	−0.132 (−0.611–0.344)
16	PBMC ^51^Cr	(71) Mawle 1997	25	26.9 ± 17.3 (26)	25.5 ± 16 (50)	1.055	−0.150 (−0.629–0.326)
17	PBMC ^51^Cr	(71) Mawle 1997	12.5	16.9 ± 11.8 (26)	17.1 ± 13 (50)	0.988	0.030 (−0.446–0.508)
18	PBMC ^51^Cr	(71) Mawle 1997	6.25	11.8 ± 7.1 (26)	10.4 ± 6.1 (50)	1.135	−0.092 (−0.570–0.385)
19	PBMC ^51^Cr	(71) Mawle 1997	3.1	6.7 ± 3.1 (26)	6 ± 4.6 (50)	1.117	−0.167 (−0.646–0.309)
20	PBMC Annexin	(79) Brenu 2014	12.5	22.4 ± 10.4 (30)	25.7 ± 9.5 (25)	0.870	0.325 (−0.209–0.869)
21	PBMC Annexin	(79) Brenu 2014	25	19 ± 10.4 (30)	25.7 ± 10.5 (25)	0.741	0.632 (0.093–1.190)
22	PBMC Annexin	(79) Brenu 2014	50	18.6 ± 11.5 (30)	29 ± 16.7 (25)	0.639	0.727 (0.185–1.291)
23	PBMC Annexin	(94) Huth 2016	6.25	4 ± 3 (14)	4 ± 8.9 (11)	1.000	0.000 (−0.806–0.806)
24	PBMC Annexin	(94) Huth 2016	12.5	6 ± 8.9 (14)	7 ± 8.1 (11)	0.857	0.113 (−0.690–0.923)
25	PBMC Annexin	(94) Huth 2016	25	11 ± 9.6 (14)	14 ± 7.4 (11)	0.786	0.333 (−0.467–1.156)
26	PBMC Annexin	(80) Hardcastle 2015	12.5	10.1 ± 3.5 (23)	19.6 ± 10.2 (22)	0.519	1.230 (0.605–1.898)
27	PBMC Annexin	(80) Hardcastle 2015	25	19.6 ± 8.7 (23)	24.6 ± 13.6 (22)	0.797	0.432 (−0.158–1.038)
28	PBMC Annexin	(80) Hardcastle 2015	50	29 ± 8.7 (23)	43.5 ± 13.6 (22)	0.667	1.254 (0.628–1.925)
29	PBMC Annexin	(80) Hardcastle 2015	12.5	8.7 ± 7.7 (18)	19.6 ± 10.2 (22)	0.445	1.165 (0.506–1.873)
30	PBMC Annexin	(80) Hardcastle 2015	25	15.9 ± 12.3 (18)	24.6 ± 13.6 (22)	0.646	0.654 (0.020–1.315)
31	PBMC Annexin	(80) Hardcastle 2015	50	26.1 ± 12.3 (18)	43.5 ± 13.6 (22)	0.600	1.308 (0.638–2.032)
32	NK Annexin	(93) Brenu 2010	25	13.6 ± 5.1 (10)	34.3 ± 6.6 (10)	0.397	3.361 (2.076–4.944)
33	NK Annexin	(83) Brenu 2011	25	15 ± 12 (35)	28 ± 18 (35)	0.536	0.840 (0.357–1.342)
34	NK Annexin	(84) Brenu 2012	25	13 ± 16.1 (65)	33 ± 13.7 (21)	0.394	1.274 (0.754–1.816)
35	NK Annexin	(84) Brenu 2012	25	14 ± 16.1 (65)	32 ± 13.7 (21)	0.438	1.146 (0.632–1.681)
36	NK Annexin	(84) Brenu 2012	25	4 ± 32.2 (65)	28 ± 13.7 (21)	0.143	0.823 (0.321–1.341)
37	NK Annexin	(85) Marshall-Gradisnik 2016	25	17 ± 4 (39)	32 ± 6 (30)	0.531	2.987 (2.323–3.719)
38	NK Annexin	(86) Nguyen 2017	1	3.4 ± 2.6 (15)	8.9 ± 4.6 (25)	0.387	1.354 (0.664–2.098)
39	NK Annexin	(87) Eaton 2018	6.25	2.3 ± 8.5 (8)	2.2 ± 2.4 (9)	1.045	−0.016 (−0.999–0.966)
40	NK Annexin	(87) Eaton 2018	12.5	2.3 ± 4.2 (8)	5.5 ± 4.5 (9)	0.418	0.696 (−0.282–1.749)
41	NK Annexin	(88) Balinas 2019	25	6 ± 6 (10)	16 ± 13 (10)	0.375	0.946 (0.035–1.941)
42	NK Annexin	(89) DuPreez 2021	6.25	2 ± 4.1 (17)	6 ± 4.1 (17)	0.333	0.953 (0.254–1.697)
43	NK Annexin	(89) DuPreez 2021	12.5	6 ± 8.2 (17)	10 ± 8.2 (17)	0.600	0.476 (−0.204–1.180)
44	NK Annexin	(89) DuPreez 2021	25	16 ± 16.5 (17)	28 ± 16.5 (17)	0.571	0.710 (0.023–1.432)
45	NK Annexin	(90) Eaton-Fitch 2021	6.25	4 ± 1.9 (15)	7 ± 5.8 (15)	0.571	0.676 (−0.054–1.445)
46	NK Annexin	(90) Eaton-Fitch 2021	12.5	9 ± 3.9 (15)	13 ± 7.7 (15)	0.692	0.638 (−0.092–1.402)
47	PBMC ^51^Cr LU	(72) Barker 1994	17.7	38.8 ± 26.7 (16)	119.8 ± 115.4 (12)	0.324	1.011 (0.230–1.852)
48	PBMC ^51^Cr LU	(73) Ojo-Amaize 1994	17.7	38 ± 28.3 (20)	70 ± 55.3 (50)	0.543	0.642 (0.116–1.183)
49	PBMC ^51^Cr LU	(74) See 1996 IFNa	10	89.1 ± 18.9 (15)	125.7 ± 24.7 (20)	0.709	1.595 (0.850–2.414)
50	PBMC ^51^Cr LU	(74) See 1996 IFNa	10	87.8 ± 19.6 (26)	125.7 ± 24.7 (20)	0.698	1.697 (1.038–2.415)
51	PBMC ^51^Cr LU	(75) See 1997 Echin	14.1	41.4 ± 80.9 (20)	112.7 ± 89.0 (20)	0.367	0.822 (0.184–1.493)
52	PBMC ^51^Cr LU	(76) See 1998 Glyco	14.1	42.3 ± 141.2 (91)	114.7 ± 151.2 (30)	0.369	0.501 (0.085–0.923)
53	PBMC ^51^Cr LU	(77) See 1998 Homeo	12.6	47.3 ± 72.4 (20)	103.7 ± 98.8 (20)	0.456	0.638 (0.007–1.295)
54	PBMC ^51^Cr LU	(78) Levine 1998	17.7	19.9 ± 5.1 (8)	83.6 ± 50.4 (8)	0.238	1.681 (0.581–2.976)
55	PBMC ^51^Cr LU	(91) Whiteside 1998	17.7	54 ± 30.4 (8)	123.6 ± 133.3 (51)	0.437	0.548 (−0.203–1.314)

**Figure 2:**
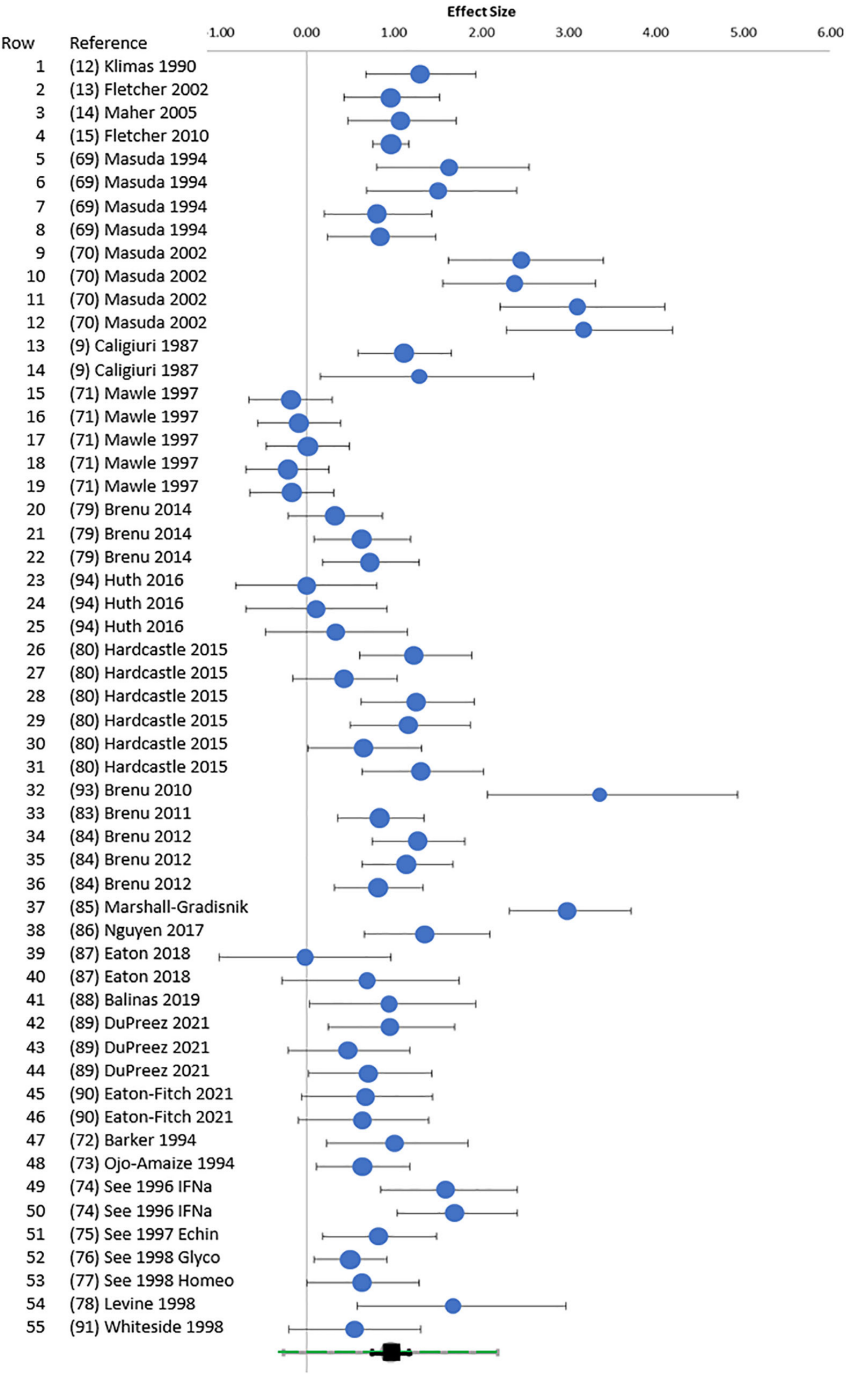
Random forest. Random forest plot. Hedges’ g (95% CI) was plotted for each E:T ratio for points cross-referenced to **Table 1** (rows 1–55). The green square at the bottom was the overall Hedges’ g with 95% CI (black) and 95% prediction interval (green).

Row refers to **Figure 2**. Mean ± SD (number). CI, 95% confidence interval. E:T, NK Effector:target K562 cell ratio; WB, whole blood; ^51^Cr, 51 Chromium method; PBMC, peripheral blood mononuclear cells; Annexin, fluorescent cytometry for Annexin V binding to apoptotic K562 cells; NK, purified natural killer cells.

The literature search completed by JNB for this meta-analysis on 1 January 2024 yielded 522 records from Embase (n=300), Scopus (n=57), and PubMed (n=165) databases. The search identified eight new references published after 2018 plus nine older studies not previously found for a total of 17 additional NK cell cytotoxicity records. One foreign article and two abstracts were found. References, related articles, and cited literature were examined for any additional pertinent information. The process yielded 58 relevant records.

The database search was repeated in PubMed and Google Scholar on 21 March 2024 and identified 498 records. Applying ME/CFS and cytotoxicity filters decreased the list to 244. Removing duplicates (n=67) and papers that did not report on NK cell cytotoxicity (n=121) or other criteria reduced this list to 25 publications. Papers published since 2019 and three older papers were found that had not been previously retrieved. Conversely, three studies found by the 1 January 2024 search were not found by the later database review. This emphasized the need to scrutinize references to find papers that do not share keywords or mention cytotoxicity in the title or abstract yet present data in Results or **Supplementary Online Materials**.

The survey results were compiled, duplicates removed, and the remaining 59 records read for quantitative NK cell cytotoxicity results from ME/CFS and healthy control subjects using fresh specimens. No relevant papers were excluded because they were behind paywalls or otherwise inaccessible. A total of 31 studies were excluded because of inadequate methods or missing data (19, 32, 40–68). The remaining 28 records (9, 12–15, 69–91) (**Table 1** and **Supplementary Table S1**) were included in the meta-analysis.

### Manuscript characteristics

The included studies were assessed for effects of cell source, method, anticoagulant, and diagnostic criteria (**Supplementary Table S1**). ME/CFS was diagnosed using 1988 Holmes (1) criteria (9, 12, 13, 69–78), 1994 Center for Disease Control “Fukuda” (2) criteria (14, 15, 79, 83–85, 87–93), and 2011 International Consensus Criteria (3, 86, 94). There was a paucity of quality of life and fatigue severity data, which limited investigation of relationships between these features of disease and NK cell cytotoxicity as a potential biomarker. Cells were collected as whole blood (12–15, 69, 70), PBMC (72–80, 91, 94), and NK cells (82–90, 93) purified by negative selection. Samples were collected via venipuncture in blood collection tubes containing the anticoagulants heparin or EDTA (20). Whole blood ^51^Cr studies were corrected for CD3-CD56+ NK cells (12–15). Isolated NK cells reported a high purity for CD3-CD56+ cells (>95%).

NK cell cytotoxicity methods were ^51^Cr release assay (9, 12–15, 45, 49, 69–78) and fluorescent cytometry of annexin V binding to cell surface phosphatidylserine as a marker of apoptosis (32, 59, 84, 87–90, 93, 94). E:T ratios ranged from 100:1 to 1:1 in the various studies with data reported for each E:T ratio, or extrapolated to theoretical ratios of 1:1 (12–15) or 50:1 (32). E:T ratios were inferred for studies reporting lytic units (LU). NK cell cytotoxicity data were presented for each E:T ratio, extrapolated E:T ratio, and LU (55 data points) (**Table 1** and **Supplementary Table S2**). Six studies employed the whole blood ^51^Cr method of which four involved extrapolation of E:T dose responses to 1:1 (12–15). PBMCs and ^51^Cr quantification were used in 10 studies (9, 33, 71–78). PBMCs and fluorescent cytometry of annexin V on target cells was used in three studies with 12 E:T ratios (79, 80, 94). Purified NK cells with fluorescent cytometry of annexin V were assessed at 15 E:T ratios in nine studies (83–90, 93).

There were diverse reasons for study exclusion. Two studies found equivalent cytotoxicity in ME/CFS and HC but did not report the data (40, 41). Nine records reported significantly reduced cytotoxicity in ME/CFS, but six did not include control subjects (19, 43–45, 65, 68) and four did not provide any quantitative data (46–48, 66). Five cytotoxicity studies did not use accepted definitions of ME/CFS but instead defined subjects as low NK cell syndrome (51–54) and EBV infection (50). Seven studies did not report cytotoxicity (55–60, 67). Three papers were excluded for technical reasons including using cells that had been frozen or shipped overnight (32, 45, 49) and measurement of cytoplasmic LDH release into the supernatant (42). Four other papers did not meet the search criteria (61–63).

### NK cell cytotoxicity in ME/CFS

Overall, Hedges’ g was 0.96 (0.75–1.18) (95% confidence interval) for the entire set of 28 studies, 2,982 subjects, and 55 E:T ratios using the random effects model (**Supplementary Table S3**) (35–37). The random forest plot showed the range of effect sizes for whole blood extrapolated to 1:1, LU, and individual E:T ratio data points (**Figure 2**). The data were explored to examine factors that may have influenced the effect size outcome (95). In general, effect sizes for methods were ranked whole blood ^51^Cr [1.60 (1.06–2.14)] > NK annexin [1.08 (0.63–1.53] > PBMC annexin [0.70 (0.41–0.98)] = PBMC ^51^Cr (**Table 2** and **Supplementary Table S3**).

**Table 2:** Hedges’ g. Cytotoxicity levels were compared between ME/CFS and HC for bins of E:T ratios in annexin dose responses and ^51^Cr assays.

Test	E:T	Hedges’ g	ME/CFS	HC	2t unp t	ME/HC	N
NK Annexin	6:1	0.79 (0.25–1.34)	2.9 ± 0.9	6.0 ± 2.8	0.082	0.485	4
NK Annexin	12:1	0.58 (0.45–0.71)	5.8 ± 3.4	9.5 ± 3.8	0.27	0.607	3
NK Annexin	25:1	1.41 (0.74–2.08)	12.3 ± 4.7	30.7 ± 7.5	4.2E−05	0.402	8
PBMC Annexin	12:1	0.59 (0.07–1.10)	10.2 ± 7.2	15.2 ± 9.2	0.37	0.675	5
PBMC Annexin	25:1	0.54 (0.39–0.68)	16.4 ± 3.9	22.2 ± 5.5	0.13	0.737	4
PBMC Annexin	50:1	1.06 (0.68–1.44)	24.6 ± 5.4	38.7 ± 8.4	0.070	0.635	3
WB ^51^Cr 1:1	1:1	1.00 (0.90–1.11)	15.0 ± 5.5	34.8 ± 11.2	0.019	0.432	4
Masuda WB ^51^Cr (70)	10:1, 20:1	1.95 (1.30–2.60)	9.7 ± 4.8	29.2 ± 5.4	2.4E−06	0.331	8
Caligiuri PBMC ^51^Cr (9)	30:1, 60:1	1.15 (1.01–1.29)	31.5 ± 7.8	52.0 ± 8.5	0.00013	0.606	2
LU PBMC ^51^Cr	LU	0.94 (0.62–1.26)	51.0 ± 23.1	108.8 ± 19.8	3.3E−05	0.473	9
Mawle PBMC ^51^Cr (71)	5 ratios	−0.13 (−0.21–−0.04)	20.4 ± 13.0	17.7 ± 9.9	0.72	1.152	5

Hedges' g values were binned for E:T ratios and values estimated using Meta-Essentials (35–37). Mean ± SD, [95% CI], 2 tailed unpaired Student’s t-test.

**Figure 3:**
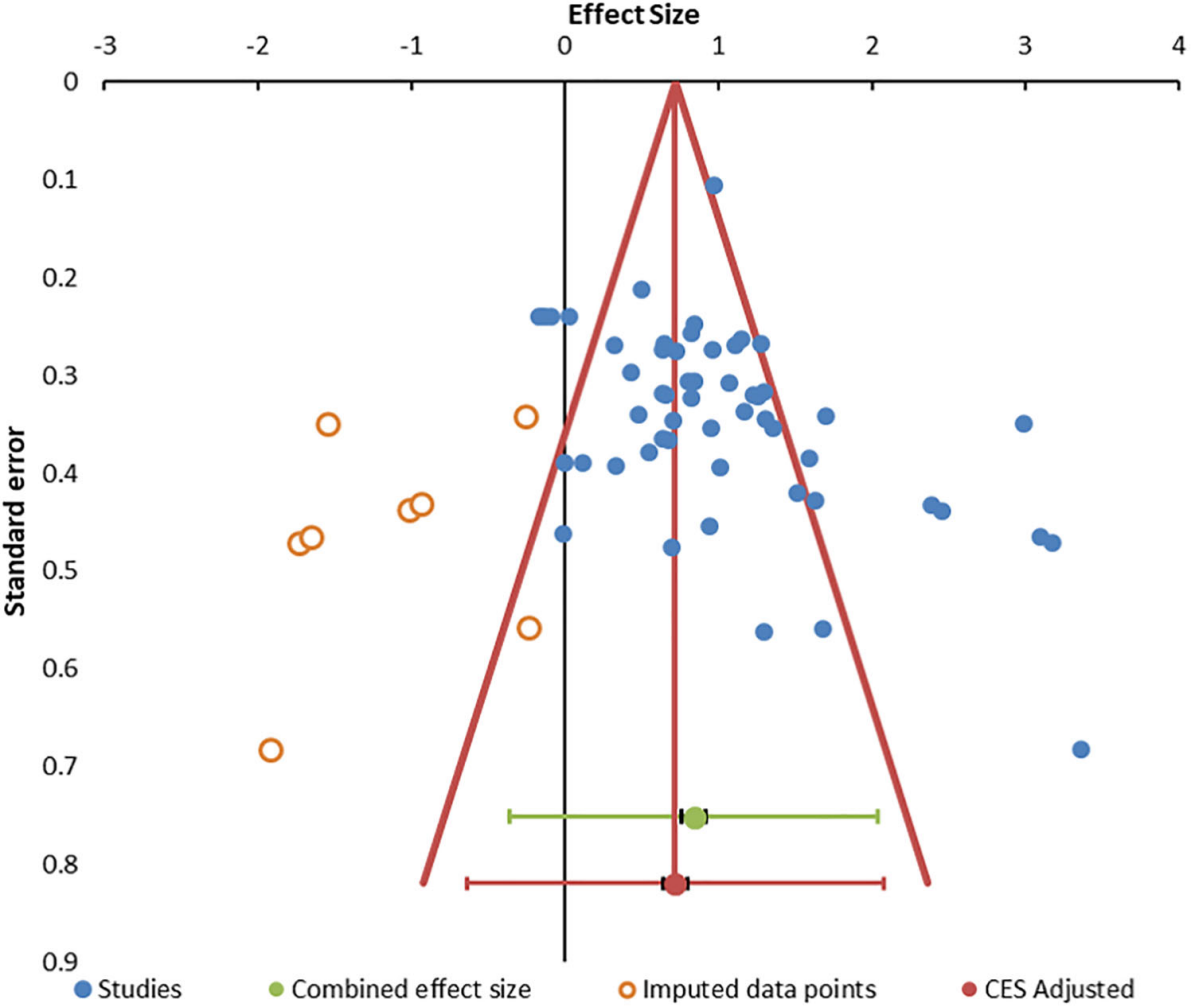
Funnel. Funnel plot for publication bias. The 55 data points are indicated by blue circles. Imputed data points are open red circles. The combined effect size (green circle), confidence interval, and prediction intervals are shown in green. The adjusted combined effect size is red.

Heterogeneity was present based on the wide distribution of Hedges’ g values in the random forest plot (**Figure 2**), funnel plot (**Figure 3**), *I^2^* of 80.03, and Q of 270.45. The high heterogeneity was likely due to low cytotoxicity at low E:T ratios, outliers with high g (70, 85, 93), and one apparently negative study that contributed five E:T ratios with Hedges’ g of 0.02 to −0.18 (71).

The distribution of outliers was assessed by standardized residual histogram that was symmetrical with skew of 0.309, suggesting equal balance of high and low effect sizes around the mean but was squat (kurtosis = 0.606) with significant outliers at both ends of the curve (**Supplementary Figure S1**) (96, 97). The Galbraith plot reiterated the pattern of outliers. The quantile plot was linear (y = 2.258x + 0.242; R² = 0.969) suggesting a single population of studies (98). Hedges’ g was not related to sample size by regression analysis (R^2^ = 0.01), indicating that studies with small sample sizes did not bias the outcome (99). Data for these plots are in **Supplementary Table S2**.

Publication bias was suggested by the funnel plot (**Figure 3**) (96, 99) and significant Egger (p = 0.014) and Begg and Mazumdar (p = 0.001) tests (**Supplementary Table S4**). The trim and fill method predicted eight missing studies (**Supplementary Table S5**). The method of Gleser and Olkin (100) predicted that 53 unpublished studies (i.e., E:T ratios) would be needed to overcome the published literature (**Supplementary Table S6**). Rosenthal’s Failsafe N of 9,556 (z = 21.74) (101) and Fisher Failsafe-N of 3,571 (Fisher’s chi-squared p<0.0001) (102) suggested that large numbers of unpublished data would be required to negate the current meta-analysis. Reiteration to account for these influences led to an adjusted combined effect size of 0.75 (95% CI, 0.67–0.83).

Additional investigations found no differences based on number of subjects in each study, diagnostic criteria, anticoagulant, cell source, method, or E:T ratio by moderator analysis and univariate general linear modeling. Year of publication was not significant, which was in agreement with the consistency of the ^51^Cr and annexin methodologies over time. Estimated marginal means in univariate analysis with these variables found that effect sizes were larger in whole blood (WB) [1.53 (1.09–1.98), p = 0.00051] and NK [1.14 (0.79–1.50), p = 0.0081] than PBMC [0.44 (0.094–0.79)]. Variances were homogeneous between studies (Levene test p > 0.15). Effect sizes exhibited heteroskedasticity (p = 0.028 by the modified Breusch–Pagan test). There were insufficient data to infer effects of age, duration of disease, gender, fatigue severity, or disability.

**Figure 4:**
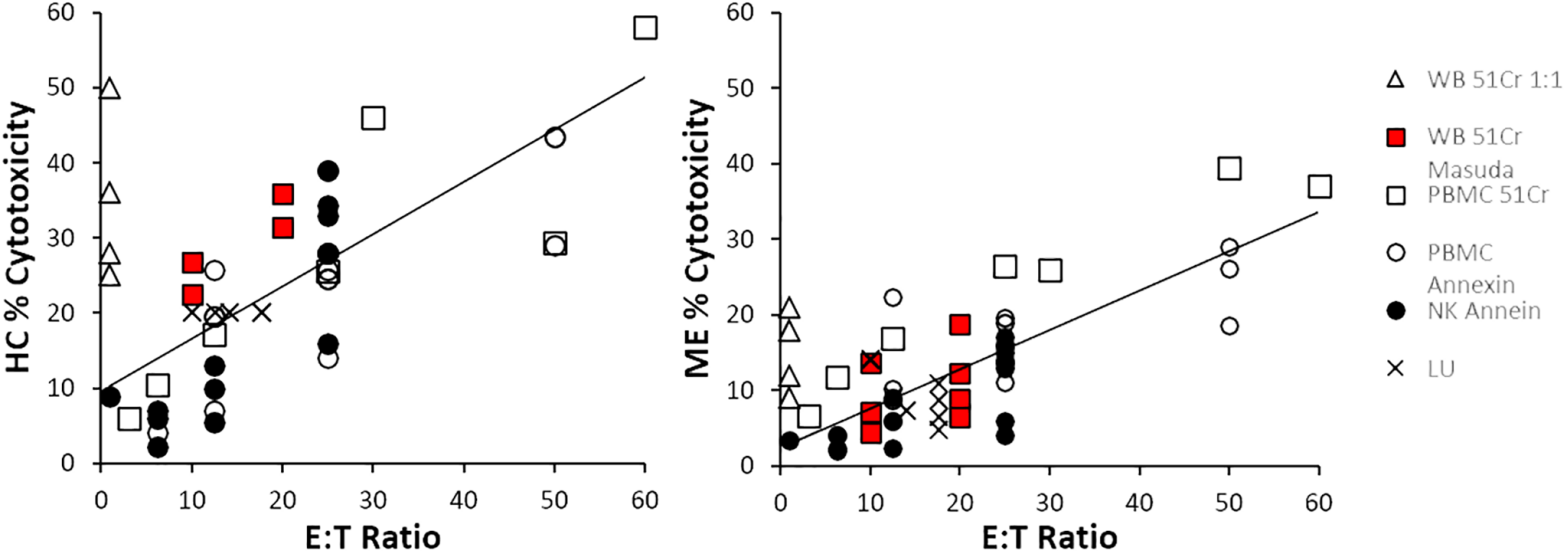
Cytotoxicity and E:T ratio. The correlations between cytotoxicity and E:T were determined for all data points with the exception of whole blood ^51^Cr extrapolated to 1:1, which were outside the experimental testing range of E:T ratios (open triangles). The slopes of the regression line from the other whole blood ^51^Cr tests (red squares), PMBC ^51^Cr (open squares), PBMC annexin (open circles), NK cells with annexin (black circles), and lytic units (LUs, crosses) were higher in HC than ME/CFS.

The relationship between % cytotoxicity of ME/CFS and HC groups at each E:T ratio was examined by scatter plot. ME/CFS NK cell cytotoxicity was approximately half that of HC (**Figure 4**). Data from each combination of cell source and method were distributed along the regression lines. There was a strong correlation (R^2^ = 0.75) when LU data were plotted as the published % cytotoxicities (**Supplementary Figure S2**). Data from four whole blood ^51^Cr experiments that were extrapolated to 1:1 (open triangles) (12–15) were not used for the regressions because the 1:1 ratio was outside the range of ratios tested in the experiment and were not comparable to the other E:T data. NK cell cytotoxicity correlated with E:T ratios for HC (R^2^ = 0.57) and ME/CFS (R^2^ = 0.61). Data points for cytotoxicity <10% and E:T ≤ 12.5:1 were closely clustered and may not discriminate between HC and ME/CFS.

The linear relationship was studied further by plotting the ratio of ME/CFS to HC cytotoxicity against E:T (**Supplementary Figure S3**). In general, there was no correlation. The majority of the ME/CFS to HC values were between 0.15 and 0.8 (0.57 ± 0.26, mean ± SD, n=55). The mean plus on SD was 0.828, which may be serve as an upper limit of % cytotoxicity in ME/CFS.

There were no correlations between effect size and E:T ratio when all data were plotted (**Supplementary Figure S4**).

Receiver operating characteristics (ROC) investigated the optimal range for NK cell cytotoxicity % across assays. ROC were computed for % cytotoxicities of ME/CFS and HC for each cell source, method, LU, bins of E:T ratios, and values extrapolated to 1:1 to provide guidance for thresholds that would discern ME/CFS from HC. A general trend was the increase in threshold with increasing E:T ratios. The ratio of % cytotoxicity over E:T was taken as a proxy for killing per single NK cell and was 0.775 (0.317–0.777] and HC 1.45 (1.05–1.84) and had an ROC threshold of 0.84. ROC thresholds were calculated for individual methods (**Supplementary Table S7**). When all data were assessed, the overall threshold for % cytotoxicity was 17.1% with sensitivity and specificity of 0.764 and corresponded to an E:T ratio of approximately 25:1 (**Table 3**).

The average cytotoxicities for ME/CFS and HC at binned E:T ratios were presented to place the ROC (**Table 3**) and effect size (**Table 2**) data into perspective.

**Table 3:** ROC for E:T ratios.

E:T ratios	N	AUC	Significance	Threshold	Sensitivity	Specificity
6.25: 1	7	0.673	0.26	5	0.714	0.714
12.5: 1	21	0.841	<10^−10^	13.8	0.765	0.765
25: 1	17	0.905	<10^−10^	19.3	0.846	0.846
50: 1	6	0.875	<10^−10^	31	0.667	0.667
WB 1:1 extrapolated	4	1	<10^−10^	23	1	1
LU	9	0.951	<10^−10^	85.7	0.778	0.778
All E:T	55	0.704	<10^−10^	17.1	0.764	0.764
% cytotoxicity E:T	42	0.789	<10^−10^	0.84	0.786	0.786

Receiver operating characteristics for % cytotoxicity were calculated for bins of E:T ratios, LU whole blood, and ratio of ME and HC %cytotoxicity/E:T ratio (**Table 1**). ME/CFS % cytotoxicity at and below the threshold would be abnormal compared to HC by nonparametric statistics.

## Discussion

NK cell cytotoxicity in ME/CFS groups was approximately half that of HC (**Figure 4**). The difference had a large effect size with Hedges’ g of 0.96 (0.75–1.18), indicating that the measurement is a reproducible biomarker despite differences in assay methods. The high heterogeneity was explained by low % cytotoxicity at low E:T and high and low outliers. The diversity of research methods complicates direct comparisons between studies and emphasizes the need for standardized protocols in future research.

There is no consensus in the literature for the range of normal for NK cell cytotoxicity and levels that indicate “significant” loss of cytotoxicity. Research practice has been to compare ME/CFS to HC cohorts. This strategy is difficult to convert to a viable clinical laboratory test because of the need for a parallel HC population and algorithms to deal with confounding conditions such as cancer, HIV, obesity, and congenital reductions in NK cell activity. One alternative has been to set the mean of the HC group minus one SD as the lower threshold of normal to account for 80% of the normal distribution (33, 78, 91). Conversely, the mean and one SD for the ME/CFS to HC ratios accounted for 83% of ME/CFS cytotoxicity (**Supplementary Figure S3**). The distribution for Hedges’ g ± one SD identified the high studies (70, 85, 93) and low outliers (71, 79, 87, 94) that generated the large study heterogeneity. ROC analysis calculated thresholds for cytotoxicity reached a consensus of 17.1% for E:T ratios of 25:1, 23.0 for WB ^51^Cr results extrapolated to 1:1 (12–15), and 18.6% for all other ^51^Cr results including LU (**Table 3** and **Supplementary Table S7**).

The meta-analysis suggests that the ^51^Cr whole blood assay remains the gold standard (**Figure 2**). However, limitations remain due to the spontaneous ^51^Cr release from the target cells that will increase the background radioactivity and reduce the signal-to-background ratio over time. This sets a practical limit to the incubation time of ^51^Cr measurements (103). The method of cell lysis for the determination of total cellular ^51^Cr must be stated as detergent solubilization release more than hypotonic lysis. The potential health effects, specific skill set, and laboratory radiation safety requirements pose additional challenges. Fluorescent cytometry with NK cells at E:T of 25:1 and higher doses (**Figure 3**) is a viable non-radioactive method that correlates with ^51^Cr release (104, 105).

Purified NK cells provide a direct assay of cytotoxicity without other cellular or plasma interactions. Whole blood includes plasma, circulating cytokines, hormones, and other factors that help reflect the *in vivo* milieu, and platelets, erythrocytes, granulocytes, and lymphocytes that may interact or interfere with NK cell function. Transferable factors such as autoantibodies have been considered as pathological agents in ME/CFS that could be responsible for inhibiting NK cell function. However, removing plasma for PBMC and NK cell purification did not alleviate the deficit in ME/CFS. Studies of PBMCs using ^51^Cr and annexin had comparable effect sizes, suggesting that the two methods were equivalent.

Alternative methods may be introduced to decrease the number of NK cells required and simplify protocols for routine clinical laboratory use. Many variations have been proposed but not studied in ME/CFS including using 500 instead of 5,000 target cells per well in ^51^Cr assay (107), diverse labeling agents for target cells including calcein-acetoxymethyl diacetylester (calcein AM) (108) and europium (109, 110), combination of DNA staining SYTOX Green with Annexin V for apoptosis (111), and non-invasive, quantitative image-based cytometry (112) and newer instruments (113). Technological advances such as microfluidic devices may allow high throughput assays for highly reproducible robust endpoints. Individual cell analysis may reveal additional dysfunction such as cytolytic heterogeneity that may contribute to ME/CFS pathology. For example, only 20% of NK cells were reported to be highly efficient killers, suggesting another variable to take into consideration (114–116). The number of K562 cells killed per NK cell is lower in ME/CFS [0.11 (0.05–0.23) (95%CI)] than HC [0.64 (0.36–1.32) (95%CI)] (12), suggesting that the efficient killers may lyse several target cells during the 4-h incubation period while the majority may not contribute. A spectrum for killing efficiency may relate to differences in NK cell phenotypes between tissue-based cytokine-producing CD3-CD56+ that are approximately 10%–20% of peripheral blood NK cells and blood-borne CD3-CD56dimCD16+ and CD3-CD56dimCD16+C57+ cells with greater cytotoxic proficiency. Future studies should enumerate the phenotypes and estimate separate E:T ratios for each. Not adjusting for NK cell phenotype may explain the relatively poor cytotoxicity for PBMCs (**Figure 4**). Decreasing the yield of CD3-CD56dimCD16+ NK cells is another reason to avoid freezing (106).

Measuring NK cell degranulation is another alternative, as this function is an essential component of cytotoxicity. Measures of degranulation include upregulation of NK cell surface CD107 expression (58) and release of dense and light granule proteins such as perforin, granzyme, chemokines, and interferon gamma into supernatants (117). The frequency of upregulation of CD107a expression as a measure of degranulation was twice the rate of cytotoxicity (45). Degranulation assesses stages from membrane contact of NK cells and target cells to granule release and upregulation CD107a and other surface receptors but does not assess the killing function and induction of apoptosis in the target cell with upregulation of annexin V and entry of dyes that stain DNA (118). Studies of degranulation alone were excluded because they do not fully assess mechanisms of cell killing. Studies of antibody-dependent cellular cytotoxicity (ADCC) were also excluded (67) because the method depends on cellular targets coated with exogenous antibodies and activation through CD16. The mechanism of activation is different, although later stages may utilize many of the same pathways as natural cytotoxicity.

The sample condition is an important consideration in the experimental design. Freezing cells and overnight shipping of blood or PBMC leads to loss of cytotoxicity and, in particular, loss of the deficit in ME/CFS compared to control (45). Theorell et al. (49) was excluded because they used (a) frozen cells even though frozen NK cells do not preserve cytotoxicity after being thawed and (b) a non-standard method of quantification that did not report E:T ratios. Querec et al. (32) used shipped cells and measured cytotoxicity by intracellular DNA staining that does not provide a suitable measure of early apoptotic cells (111). It is well reported that NK surface marker expression and cytotoxicity are poorly preserved after delayed use and freezing (33, 106, 119–121). NK cell cytotoxicity is reduced after freezing overnight in ^51^Cr assays of whole blood and PBMC with 26.2% ± 21.6% cytotoxicity when fresh dropping to 5.0% ± 3.1% after freezing (45). The levels of cytotoxicity between fresh and frozen cells were correlated. The physiological and cellular cause(s) of the deficient NK cell cytotoxicity in ME/CFS and the loss of % cytotoxicity with storage, remain unknown. The use of specimens after prolonged shipping or freezing and thawing is discouraged until reliable cryopreservation methods are validated. Freshly isolated samples are advocated for higher sensitivity and accuracy of cytotoxicity measurements. New methods will be needed in the future to maintain NK cell function during shipping to central laboratories for approved clinical testing.

K562 cells were the targets for cytotoxicity testing. Studies with EBV-transformed and other tumor cell lines were excluded. Studies using non-specific PMA, PHA, and concavalin stimulation or other measures of immune dysregulation in CFS were excluded (43, 55, 80, 81, 89, 122–126). Culture with IL2, IL15, and IL18 can increase cytotoxicity by up to 10-fold (43, 89). However, cytokine-cultured cells were excluded because they develop “cytokine-induced cytotoxicity” (125, 126) and lymphokine-activated killer (LAK) cell phenotypes *in vitro* that do not reflect NK cell function *in vivo*. A complicating factor is that blood NK cells have a higher proportion of CD56+CD16+ NK cells with greater cytotoxic potential in contrast to tissues that contain more CD56^Bright^ NK cells that may have different functions and roles in disease. This issue will remain to be addressed in the future.

Mechanisms for decreased cytotoxicity may involve increased expression of inhibitory KIR or checkpoint proteins, increased SHP1 expression that suppresses kinase pathways that are essential for degranulation and cytotoxicity, reduced intracellular dense or light granule proteins, decreased chemokine, TNFalpha or INFgamma production, suppression of mTOR and glycolysis, or other metabolic and energetic alterations. Investigation of degranulation issue is relevant to other degranulating cells such as CD8 T lymphocytes and presynaptic neurons that share many of the same pathways and proteins in shuttling granules for exocytosis. Therefore, studies in NK cells may shed light on important immune deficits of host defense and the cognitive dysfunction of ME/CFS. This line of reasoning highlights the case that NK cells may be a model system for studying the molecular pathogenesis of ME/CFS. Drug testing in the NK cell model *in vitro* is possible given past results with inosine (44), INFalpha (74), various supplements (75–77), glucagon-like peptide 1 (127), and naltrexone (90, 128, 129). The current outcomes support those innovations and provide effect sizes to help design future investigations.

The differential diagnosis of low NK cytotoxicity is of importance in considering the specificity for ME/CFS. Low NK syndrome with chronic fatigue was proposed by Aoki et al. (51, 52) and investigated in EBV (50) and families of ME/CFS patients (78). NK activity was reproducible over time in these studies (54, 130) and ME/CFS (84). NK cell cytotoxicity may decrease after 75 years of age (131). Genetically defined congenital immunodeficiency with <1% NK cells in blood has been associated with mutations of GATA2 and MCM4, while functional loss of antibody-dependent cellular cytotoxicity is associated with FCGR3A abnormalities (132). NK cell numbers and function are reduced in HIV infection (75). These diseases emphasize the need for history and physical and laboratory examination to rule out treatable and other diagnosed conditions in order to make the diagnosis of ME/CFS. Routine evaluations will prevent false positive attributions of reduced NK cell numbers or function due to major depression, alcohol abuse, neoplasia, HIV, and chronic infectious and autoimmune diseases. Hospitalization for acute severe depression was associated with reduced NK cell cytotoxicity (133, 134). The defects resolved after 6 months of successful treatment, suggesting that neuroendocrine alterations may be capable to induce NK cell dysfunction. Hospitalization per se and schizophrenia were not related to the immune deficit (135). Chronic alcohol abuse reduced cytotoxicity and had an additive effect with depression (136). The interaction of male gender and major depression may cause more significant deficits in NK cell function in contrast to women (134). These conditions are standard exclusions for ME/CFS diagnosis (137–140). Studies of interactions of these variables and ME/CFS could not be done using currently available data, but may be accessible at the individual level if common data elements for ME/CFS severity, quality of life patient-reported outcome measures, depression, and other relevant measures are included in future studies (32, 43, 79, 90) and used in multivariate analyses.

The study of Mawle et al. (71) contributed five E:T data points and appeared to be an outlier in the meta-analysis because NK cell cytotoxicity was equivalent between ME/CFS and HC. The authors reported no differences by subgroup analysis for sudden or gradual onset, duration of illness, or health status. Criticism of this study (71) noted that subjects with ME/CFS for longer than 10 years were excluded from recruitment (19), and frozen blood from the ME/CFS subjects did not show evidence of active or latent infection by herpes virus 6 (HHV-6) (141) in contrast to other studies (142–144). This study was considered a potential outlier for heterogeneity analysis.

Obesity reduces NK cell cytotoxicity (145–148). However, few studies reported BMI or excluded obese ME/CFS or control subjects (90), meaning that this variable cannot be addressed retrospectively. Hyperlipidemia, peroxisome proliferator-activated receptor (PPAR) alpha/delta agonist drugs, butyrate (149), and other metabolic changes in obesity reduce the function of mammalian target of rapamycin (mTOR) and NK cytotoxicity *in vivo* and *in vitro* (150). The molecular mechanisms may be relevant to ME/CFS pathophysiology.

Literature selection bias was a concern because the heterogeneity analysis suggested the possible underrepresentation of studies reporting equivalent NK cell cytotoxicity between ME/CFS and HC. The studies that were excluded were reviewed for additional insights into publication bias. Nine records stated that NK cell cytotoxicity in ME/CFS was significantly reduced relative to historical controls (19, 43–45, 65, 68) but did not provide quantitative data (46–48, 66). Five studies reported on “low NK syndrome” subjects with low cytotoxicity and symptoms attributable to post-EBV infection status and chronic fatigue that did not meet standard diagnostic criteria (50–54). These reports document a wide range of cytotoxicity in control cohorts and makes it necessary that parallel control groups be tested to define abnormal results. In contrast to these 14 potentially positive studies, only three studies reported equivalent NK cell cytotoxicity in ME/CFS and HC. However, they were excluded for not reporting the data (40, 41) or used the insensitive LDH release assay at E:T of 100:1 (40–42). If these publications are representative of missing “file drawer” studies (101), then any unpublished data would likely continue to support the deficit in NK cell cytotoxicity of ME/CFS.

Additional limitations included concerns that the surveyed manuscripts are not representative of the field. However, the current search extended the result of prior reviews of NK cell cytotoxicity in ME/CFS (10, 19) without finding additional foreign literature or obscure negative results.

Heterogeneity was high but likely due to small effect sizes at low E:T ratios for PBMC and NK studies, especially large effect sizes in three studies (70, 85, 93) and apparently negative outcomes from Mawle et al. (71).

It is not always possible to control for population-based confounding variables while synthesizing data for a meta-analysis further biasing data. Data were stratified in order to clarify effects of diagnostic criteria, anticoagulant, type of cells, method, and E:T ratios. Data have been published as the E:T dose response, extrapolated to 1:1 or 50:1, or as lytic units (LU), which presents a challenge for comparing study outcomes. This was circumvented by comparing results for each E:T ratio. Lytic units were discouraged because they were standardized to 20% cytotoxicity unlike all of the other studies. Expressing the data as E:T allowed investigation of dose responses that were not evident by extrapolation strategies. This exposed the stratification between PBMC ^51^Cr and annexin methods, NK annexin, and whole blood ^51^Cr methods (**Tables 2**, **3** and **Figure 4**). Variables such as criteria, cells, and methods were assessed as fixed factors to determine their impact. Unfortunately, there were insufficient data to investigate age, gender, fatigue, disability, and other clinical outcomes. Alternative modeling may be of value, particularly if NK cell cytotoxicity, quality of life, and other questionnaire data from individuals were used.

NK cell dysfunction has been reported in long COVID (151) and Gulf War Illness (122, 152) that have similar symptom profiles. It will be of interest to contrast molecular mechanisms underlying loss of NK cell cytotoxicity between these conditions.

## Conclusion

NK cells from ME/CFS subjects have significantly lower cytotoxicity than control subjects. The reduction in K562 cell killing by fresh NK cells remains one of the most promising potential biomarkers for ME/CFS. Frozen and shipped cells do not retain sufficient cytotoxicity. Whole blood ^51^Cr assays have the largest effect size, but extrapolating without showing the raw data reduced the information that can be gained. Purified NK cells with E:T of 25:1 and detection by fluorescent cytometry using Annexin V for early and late apoptosis was a reasonable non-radioactive alternative. Hedges’ g and thresholds for ME/CFS and HC % cytotoxicity at various E:T values and different cell sources and methods provide guidelines to diagnose ME/CFS in future studies. Fresh specimens or new methods will be necessary for NK cell cytotoxicity to become a routine clinical laboratory test for diagnosis. Technical problems related to the assay methods are a limitation that may be overcome by innovative engineering. Future studies should report NK cell cytotoxicity with subjective common data elements to understand behavioral correlations and investigate interactions with dysfunction of metabolomics, mitochondria, and brain cell function using magnetic resonance imaging (153) in order to gain a better understanding of integrated disease pathophysiology and symptom generation. NK cells represent a model system to understand molecular mechanisms of disease in ME/CFS and for testing potential drugs *in vitro* (44, 68, 74–77, 90, 128, 129) for efficacy before human clinical trials. The effect sizes calculated here may allow improved design for future studies of deficient NK cell cytotoxicity in ME/CFS.

## Method

This meta-analysis was undertaken according to Cochrane review guidelines and aimed to identify observational studies of NK cell cytotoxic activity in ME/CFS and HC research participants for quantitative analysis.

### Database and literature search

The current analysis was built on previous systematic literature searches into NK cell function in ME/CFS carried out by Natelson in 2002 (18); Strayer, Scott, and Carter in 2015 (19); and Eaton-Fitch et al. in 2019 (10). Strayer, Scott, and Carter searched for publications in English in 2015 on PubMed and in “Google” using key words “Natural Killer Cell Activity” (NK Cell Activity), “chronic fatigue syndrome” (CFS), “Flow Cytometry,” and “Chromium 51” (19). Eaton-Fitch et al. (10) screened publications in PubMed, Medline (EBSCOhost), Embase, and Scopus for full-text terms “chronic fatigue syndrome” OR “myalgic encephalomyelitis” OR “ME/CFS” AND “natural killer cell” and medical subject headings (MeSH) for “chronic fatigue syndrome/myalgic encephalomyelitis” [including systematic exertion intolerance disease (SEID)] and “natural killer cells” (10). Papers from Natelson were cited in the more recent review.

The protocol was compared with published listings on the PROSPERO website (National Institute for Health Research) (https://www.crd.york.ac.uk/prospero/) for duplication and prospectively registered on the database (ID: CRD42024542140). An updated search of PubMed, Embase, and Scopus databases followed Preferred Reporting Items for Systematic Reviews and Meta-analyses (PRISMA) guidelines and was completed 1 January 2024 by JNB. Full-text search terms included “chronic fatigue syndrome” OR “myalgic encephalomyelitis” OR “ME/CFS” AND “natural killer cell cytotoxicity.”. Medical subject headings (MeSH) terms were used for chronic fatigue syndrome/myalgic encephalomyelitis (including systematic exertion intolerance disease), natural killer cells, natural killer cell function, and cytotoxicity. Proximity operators were not used during the literature search. Reference lists were checked, and citations were searched for additional publications. Unpublished literature was not searched. No additional papers were identified through alternative search databases such as Griffith University institute library or Google Scholar. The search strategy used in this meta-analysis was independently validated on 21 March 2024 by NE-F.

### Selection criteria

The collected articles were initially screened at the level of titles and abstracts to include studies that reported on NK cell cytotoxicity in ME/CFS patient groups. Candidate papers were read in depth to find figures, tables, and other data reporting NK cell cytotoxicity in Results and **Supplementary Online Materials** sections and additional leads to other relevant literature. This comprehensive analysis ensured that the manuscripts fulfilled inclusion criteria for the meta-analysis:

- free full text publication available through institutional access;
- original research without duplicate publication;
- comparison of ME/CFS versus healthy control (HC) subjects;
- diagnosis of ME/CFS according to criteria including 1988 Holmes (1), 1994 Center for Disease Control (“Fukuda”) (2), Canadian Consensus Criteria (CCC) for ME/CFS (5), International Consensus Criteria (ICC) for ME/CFS (3), Institute of Medicine criteria for Systemic Exertion Intolerance Disease SEID (4), or other established criteria;
- research participants were human adults age 18 years and older;
- manuscripts detailed core information including numbers of subjects in each group;
- sample collection method with anticoagulation by heparin or EDTA (20);
- sources of cells in whole blood (WB), PBMC, or purified NK cells;
- the condition of the samples as either fresh versus frozen or stored cells;
- method of NK cell cytotoxicity analysis; and
- E:T cell ratios or lytic units (LUs).

Records were excluded from the primary analysis if the ME/CFS cohort was not compared to healthy controls. This excluded treatment studies; comparisons to other patient groups such as fibromyalgia, multiple sclerosis, chronic fatigue not meeting ME/CFS definitions; Epstein–Barr virus or HIV infection; comparisons to historical control groups; binary stratification into normal and low cytotoxicity based on local laboratory standards; cells incubated with cytokines to enhance or maintain their viability and functions; and use of frozen cells or specimens with prolonged overnight shipping.

### Quality assessment

Studies were evaluated for quality and bias using the Joanna Briggs Institute Checklist for case control studies (21). Additionally, the Downs and Black checklist was followed to assess the clarity of descriptions of outcomes and findings, reported probability outcomes, recruitment details and participant representation of populations (22, 23), and described previously (10).

### Data extraction

Figures, tables, text, and **Supplementary Materials** were assessed for individual data points, sample size (N), mean, median, standard error of the mean (SEM), standard deviation (SD), 95% confidence intervals (CI), interquartile 25% and 75% ranges (IQR), and range of % cytotoxicity. Results were converted to mean, SD, and N (24–31) for each E:T ratio or extrapolated E:T values such as 1:1 (12–15), 1:50 (32), and LU (33).

Studies extrapolating to LU represented a special case. They used dose responses with E:T from 50:1 to 6:1 but did not report cytotoxicity at each ratio. Instead, data were interpolated to 20% cytotoxicity for each subject, and the number of PBMC (effectors) extrapolated per 10e^7^ cells using the formula of Pross et al. (34). One LU was defined as the number of effector cells needed to lyse 20% of the K562 target cells (1,000 out of 5,000 targets). The numbers of effector and K562 cells at 20% cytotoxicity for HC were interpolated and converted to E:T ratios and the corresponding % cytotoxicity for ME/CFS calculated from the published LU values.

### Meta-analysis and other statistics

Effect sizes for the difference between ME/CFS and HC in each study were calculated as Hedges’ g using Meta-Essentials (35–37). In addition to the overall weighted Hedges’ g from all studies, effect sizes were calculated for individual E:T ratios, cell sources, and NK cell cytotoxicity methods. Study heterogeneity was tested by Q and *I^2^* (38, 39). Publication bias was examined by funnel plot and Failsafe N test as per the Meta-Essentials manual.

Results were displayed as % cytotoxicity for ME/CFS and HC groups, ME/HC ratio of % cytotoxicity, Hedges g,and E:T ratios. Hedges’ g for subgroups were amalgamated using Meta-Essentials software (35–37). Differences between subgroups were determined by analysis of variance (ANOVA) followed by Tukey’s honest significant difference for correction of multiple comparisons. Univariate regression of ME/HC, ME/CFS, and HC data was used to study interactions of diagnostic criteria (Holmes, Fukuda, and International) (1–3), cell sources (whole blood, PBMC, and purified NK cells), anticoagulant (heparin and EDTA), methods (51Cr, annexin), number of participants in each study, and year of publication as a measure of evolving trends in methodologies. There was insufficient data to evaluate age, gender, duration of ME/CFS, quality of life, or fatigue severity.

Receiver operating characteristics (ROC) were applied to ME/CFS and HC data for each method and E:T ratio to infer significant threshold values that may be used to optimize the assays and distinguish ME/CFS from HC.

## Data Availability

The original contributions presented in the study are included in the article/**Supplementary Material**. Further inquiries can be directed to the corresponding author.
