# Supplementary material for: Meta-analysis of natural killer cell cytotoxicity in myalgic encephalomyelitis/chronic fatigue syndrome
Source: Front Immunol. 2024 Oct 17;15:1440643. doi: 10.3389/fimmu.2024.1440643 (PMC11524851; doi:10.3389/fimmu.2024.1440643)
Supplement: Supplementary file 1 [file DataSheet1.docx]

Meta-analysis of Natural Killer (NK) cell cytotoxicity in Myalgic Encephalomyelitis / Chronic Fatigue Syndrome (ME/CFS)

James N. Baraniuk, Natalie Eaton-Fitch, Sonya Marshall-Gradisnik

**Supplemental Online Materials**

SOM Table S1. Included cytotoxicity studies.

| Reference | Method | Anti-coagulant | Criteria |
| --- | --- | --- | --- |
| (12) Klimas 1990 | WB 51Cr | Heparin | Holmes |
| (13) Fletcher 2002 | WB 51Cr | Heparin | Holmes |
| (14) Maher 2005 | WB 51Cr | Heparin | Fukuda |
| (15) Fletcher 2010 | WB 51Cr | Heparin | Fukuda |
| (69) Masuda 1994 | WB 51Cr | Heparin | Holmes |
| (70) Masuda 2002 | WB 51Cr | Heparin | Holmes |
| (9) Caligiuri 1987 | PBMC 51Cr | Heparin | Holmes |
| (71) Mawle 1997 | PBMC 51Cr | Heparin | Holmes |
| (72) Barker 1994 | PBMC 51Cr LU | Heparin | Holmes |
| (73) Ojo-Amaize 1994 | PBMC 51Cr LU | Heparin | Holmes |
| (74) See 1996 IFNa | PBMC 51Cr LU | Heparin | Holmes |
| (75) See 1997 Echin | PBMC 51Cr LU | Heparin | Holmes |
| (76) See 1998 Glyco | PBMC 51Cr LU | Heparin | Holmes |
| (77) See 1998 Homeo | PBMC 51Cr LU | Heparin | Holmes |
| (78) Levine 1998 | PBMC 51Cr LU | Heparin | Holmes |
| (91) Whiteside 1998 | PBMC 51Cr LU | Heparin | Fukuda |
| (79) Brenu 2014 | PBMC Annexin | EDTA | Fukuda |
| (80) Hardcastle 2015 | PBMC Annexin | Heparin | Fukuda |
| (94) Huth 2016 | PBMC Annexin | Heparin | International |
| (93) Brenu 2010 | NK Annexin | EDTA | Fukuda |
| (83) Brenu 2011 | NK Annexin | EDTA | Fukuda |
| (84) Brenu 2012 | NK Annexin | EDTA | Fukuda |
| (85) Marshall-Gradisnik 2016 | NK Annexin | EDTA | Fukuda |
| (86) Nguyen 2017 | NK Annexin | EDTA | International |
| (87) Eaton 2018 | NK Annexin | EDTA | Fukuda |
| (88) Balinas 2019 | NK Annexin | EDTA | Fukuda |
| (89) DuPreez 2021 | NK Annexin | EDTA | Fukuda |
| (90) Eaton-Fitch 2021 | NK Annexin | EDTA | Fukuda |

SOM. Table S2. Data for included studies. Data from each paper was processed to give percent cytotoxicity for each E:T ratio and method then reported for ME/CFS and HC as Mean, SD and number of participants. Studies were tabulated as whole blood 51Cr (WB 51Cr), PBMC 51Cr, PBMC annexin, NK annexin and PBMC 51Cr Lytic Units (LU). Individual rows show each E:T ratio and correspond to the random forest plot (Figure 2). Hedges’ g between ME/CFS and HC was determined by Meta-Essentials software (35–37) for each E:T ratio. Hedges’ g was reported with 95% CI, standard error and % weight for all 55 specimens. Metadata for heterogeneity and publication bias analysis were included for each data point. CI, 95% confidence interval. E:T, NK Effector : Target K562 cell ratio; WB, Whole blood; 51Cr, 51 Chromium method; PBMC, peripheral blood mononuclear cells; Annexin, fluorescent cytometry for Annexin V binding to apoptotic K562 cells; NK, purified natural killer cells.

| Row | Method | Reference | Anti-coagulant | Criteria | E:T | ME mean | ME SD | ME n | HC mean | HC SD | HC n | ME/HC | Hedges' g | 95% CI Lower limit | 95% CI Upper limit | Weight | Standard Error | Stand-ardized residual | Inverse standard error | Z-value | Normal Quantile | Sample Quantile |
| --- | --- | --- | --- | --- | --- | --- | --- | --- | --- | --- | --- | --- | --- | --- | --- | --- | --- | --- | --- | --- | --- | --- |
| 1 | WB 51Cr | (12) Klimas 1990 | Heparin | Holmes | 1 | 9 | 9.5 | 13 | 25 | 12.6 | 69 | 0.36 | 1.301 | 0.684 | 1.942 | 0.019 | 0.316259 | 1.474192 | 3.16197 | 4.112755 | 0.742423 | 1.474192 |
| 2 | WB 51Cr | (13) Fletcher 2002 | Heparin | Holmes | 1 | 21 | 10.5 | 29 | 36 | 19 | 29 | 0.583 | 0.964 | 0.428 | 1.526 | 0.02 | 0.274104 | 0.463903 | 3.648254 | 3.517054 | 0.182205 | 0.463903 |
| 3 | WB 51Cr | (14) Maher 2005 | Heparin | Fukuda | 1 | 18 | 20 | 30 | 50 | 40 | 19 | 0.36 | 1.074 | 0.471 | 1.712 | 0.019 | 0.308213 | 0.771261 | 3.244512 | 3.484396 | 0.228475 | 0.771261 |
| 4 | WB 51Cr | (15) Fletcher 2010 | Heparin | Fukuda | 1 | 12 | 14.6 | 176 | 28 | 17.8 | 230 | 0.429 | 0.968 | 0.763 | 1.178 | 0.023 | 0.105581 | 1.333945 | 9.471369 | 9.172705 | 0.573561 | 1.333945 |
| 5 | WB 51Cr | (69) Masuda 1994 | Heparin | Holmes | 10 | 5.7 | 2.8 | 10 | 22.5 | 11.9 | 21 | 0.253 | 1.635 | 0.804 | 2.554 | 0.016 | 0.427949 | 1.870832 | 2.336725 | 3.82132 | 1.00842 | 1.870832 |
| 6 | WB 51Cr | (69) Masuda 1994 | Heparin | Holmes | 20 | 8.8 | 4.4 | 10 | 31.5 | 17.4 | 21 | 0.279 | 1.508 | 0.689 | 2.409 | 0.016 | 0.42036 | 1.601459 | 2.378914 | 3.588247 | 0.86773 | 1.601459 |
| 7 | WB 51Cr | (69) Masuda 1994 | Heparin | Holmes | 10 | 13.6 | 9.8 | 24 | 22.5 | 11.9 | 21 | 0.604 | 0.808 | 0.206 | 1.438 | 0.019 | 0.305654 | -0.10112 | 3.27167 | 2.642259 | -0.18221 | -0.10112 |
| 8 | WB 51Cr | (69) Masuda 1994 | Heparin | Holmes | 20 | 18.7 | 12.2 | 24 | 31.5 | 17.4 | 21 | 0.594 | 0.846 | 0.242 | 1.48 | 0.019 | 0.3068 | 0.024808 | 3.259456 | 2.756857 | 0 | 0.024808 |
| 9 | WB 51Cr | (70) Masuda 2002 | Heparin | Holmes | 10 | 7.2 | 4.1 | 16 | 26.7 | 9.7 | 20 | 0.27 | 2.462 | 1.628 | 3.407 | 0.016 | 0.437839 | 3.723181 | 2.283946 | 5.621958 | 1.504199 | 3.723181 |
| 10 | WB 51Cr | (70) Masuda 2002 | Heparin | Holmes | 20 | 12.3 | 3.8 | 16 | 35.9 | 12.5 | 20 | 0.343 | 2.384 | 1.561 | 3.316 | 0.016 | 0.431838 | 3.59502 | 2.315681 | 5.520514 | 1.26794 | 3.59502 |
| 11 | WB 51Cr | (70) Masuda 2002 | Heparin | Holmes | 10 | 4.4 | 2.3 | 20 | 26.7 | 9.7 | 20 | 0.165 | 3.101 | 2.222 | 4.105 | 0.015 | 0.465009 | 4.883477 | 2.150498 | 6.667829 | 1.662635 | 4.883477 |
| 12 | WB 51Cr | (70) Masuda 2002 | Heparin | Holmes | 20 | 6.5 | 2.9 | 20 | 35.9 | 12.5 | 20 | 0.181 | 3.176 | 2.286 | 4.194 | 0.015 | 0.471304 | 4.977761 | 2.121774 | 6.738176 | 1.879026 | 4.977761 |
| 13 | PBMC 51Cr | (9) Caligiuri 1987 | Heparin | Holmes | 30 | 26 | 19.2 | 41 | 46 | 15 | 25 | 0.557 | 1.114 | 0.59 | 1.664 | 0.02 | 0.268858 | 1.037306 | 3.719434 | 4.143466 | 0.370724 | 1.037306 |
| 14 | PBMC 51Cr | (9) Caligiuri 1987 | Heparin | Holmes | 60 | 37 | 16 | 8 | 58 | 14 | 6 | 0.638 | 1.293 | 0.158 | 2.605 | 0.013 | 0.561447 | 0.812374 | 1.781112 | 2.303316 | 0.275239 | 0.812374 |
| 15 | PBMC 51Cr | (71) Mawle 1997 | Heparin | Holmes | 50 | 32.5 | 16.9 | 26 | 29.4 | 17.6 | 50 | 1.105 | -0.132 | -0.611 | 0.344 | 0.021 | 0.239757 | -4.29414 | 4.170896 | -0.73694 | -1.87903 | -4.29414 |
| 16 | PBMC 51Cr | (71) Mawle 1997 | Heparin | Holmes | 25 | 26.9 | 17.3 | 26 | 25.5 | 16 | 50 | 1.055 | -0.15 | -0.629 | 0.326 | 0.021 | 0.239425 | -3.90855 | 4.176665 | -0.35183 | -1.5042 | -3.90855 |
| 17 | PBMC 51Cr | (71) Mawle 1997 | Heparin | Holmes | 12.5 | 16.9 | 11.8 | 26 | 17.1 | 13 | 50 | 0.988 | 0.03 | -0.446 | 0.508 | 0.021 | 0.239331 | -3.48653 | 4.178308 | 0.065609 | -1.37647 | -3.48653 |
| 18 | PBMC 51Cr | (71) Mawle 1997 | Heparin | Holmes | 6.25 | 11.8 | 7.1 | 26 | 10.4 | 6.1 | 50 | 1.135 | -0.092 | -0.57 | 0.385 | 0.021 | 0.239961 | -4.45097 | 4.167352 | -0.89462 | -2.25559 | -4.45097 |
| 19 | PBMC 51Cr | (71) Mawle 1997 | Heparin | Holmes | 3.1 | 6.7 | 3.1 | 26 | 6 | 4.6 | 50 | 1.117 | -0.167 | -0.646 | 0.309 | 0.021 | 0.23971 | -4.25311 | 4.171708 | -0.69579 | -1.66263 | -4.25311 |
| 20 | PBMC Annexin | (79) Brenu 2014 | EDTA | Fukuda | 12.5 | 22.4 | 10.4 | 30 | 25.7 | 9.5 | 25 | 0.87 | 0.325 | -0.209 | 0.869 | 0.02 | 0.268743 | -1.93078 | 3.721028 | 1.210165 | -1.17258 | -1.93078 |
| 21 | PBMC Annexin | (79) Brenu 2014 | EDTA | Fukuda | 25 | 19 | 10.4 | 30 | 25.7 | 10.5 | 25 | 0.741 | 0.632 | 0.093 | 1.19 | 0.02 | 0.273671 | -0.76083 | 3.654022 | 2.310451 | -0.62782 | -0.76083 |
| 22 | PBMC Annexin | (79) Brenu 2014 | EDTA | Fukuda | 50 | 18.6 | 11.5 | 30 | 29 | 16.7 | 25 | 0.639 | 0.727 | 0.185 | 1.291 | 0.02 | 0.27581 | -0.40635 | 3.625687 | 2.637254 | -0.32261 | -0.40635 |
| 23 | PBMC Annexin | (94) Huth 2016 | Heparin | Intl | 6.25 | 4 | 3 | 14 | 4 | 8.9 | 11 | 1 | 0 | -0.806 | 0.806 | 0.017 | 0.389604 | -2.16313 | 2.566708 | 0 | -1.26794 | -2.16313 |
| 24 | PBMC Annexin | (94) Huth 2016 | Heparin | Intl | 12.5 | 6 | 8.9 | 14 | 7 | 8.1 | 11 | 0.857 | 0.113 | -0.69 | 0.923 | 0.017 | 0.389931 | -1.87009 | 2.564553 | 0.289656 | -1.08684 | -1.87009 |
| 25 | PBMC Annexin | (94) Huth 2016 | Heparin | Intl | 25 | 11 | 9.6 | 14 | 14 | 7.4 | 11 | 0.786 | 0.333 | -0.467 | 1.156 | 0.017 | 0.39244 | -1.29435 | 2.548162 | 0.848484 | -0.8035 | -1.29435 |
| 26 | PBMC Annexin | (80) Hardcastle 2015 | Heparin | Fukuda | 12.5 | 10.1 | 3.5 | 23 | 19.6 | 10.2 | 22 | 0.519 | 1.23 | 0.605 | 1.898 | 0.019 | 0.320365 | 1.231067 | 3.121439 | 3.837887 | 0.469721 | 1.231067 |
| 27 | PBMC Annexin | (80) Hardcastle 2015 | Heparin | Fukuda | 25 | 19.6 | 8.7 | 23 | 24.6 | 13.6 | 22 | 0.797 | 0.432 | -0.158 | 1.038 | 0.019 | 0.296503 | -1.38158 | 3.372644 | 1.458343 | -0.86773 | -1.38158 |
| 28 | PBMC Annexin | (80) Hardcastle 2015 | Heparin | Fukuda | 50 | 29 | 8.7 | 23 | 43.5 | 13.6 | 22 | 0.667 | 1.254 | 0.628 | 1.925 | 0.019 | 0.321416 | 1.303635 | 3.111232 | 3.901392 | 0.520938 | 1.303635 |
| 29 | PBMC Annexin | (80) Hardcastle 2015 | Heparin | Fukuda | 12.5 | 8.7 | 7.7 | 18 | 19.6 | 10.2 | 22 | 0.445 | 1.165 | 0.506 | 1.873 | 0.018 | 0.337656 | 0.976093 | 2.961593 | 3.451715 | 0.322614 | 0.976093 |
| 30 | PBMC Annexin | (80) Hardcastle 2015 | Heparin | Fukuda | 25 | 15.9 | 12.3 | 18 | 24.6 | 13.6 | 22 | 0.646 | 0.654 | 0.02 | 1.315 | 0.019 | 0.319971 | -0.57988 | 3.125283 | 2.044517 | -0.46972 | -0.57988 |
| 31 | PBMC Annexin | (80) Hardcastle 2015 | Heparin | Fukuda | 50 | 26.1 | 12.3 | 18 | 43.5 | 13.6 | 22 | 0.6 | 1.308 | 0.638 | 2.032 | 0.018 | 0.344136 | 1.375506 | 2.905824 | 3.801899 | 0.627822 | 1.375506 |
| 32 | NK Annexin | (93) Brenu 2010 | EDTA | Fukuda | 25 | 13.6 | 5.1 | 10 | 34.3 | 6.6 | 10 | 0.397 | 3.361 | 2.076 | 4.944 | 0.011 | 0.682525 | 3.702759 | 1.465149 | 4.92449 | 1.376475 | 3.702759 |
| 33 | NK Annexin | (83) Brenu 2011 | EDTA | Fukuda | 25 | 15 | 12 | 35 | 28 | 18 | 35 | 0.536 | 0.84 | 0.357 | 1.342 | 0.021 | 0.246838 | 0.008891 | 4.05124 | 3.404755 | -0.04532 | 0.008891 |
| 34 | NK Annexin | (84) Brenu 2012 | EDTA | Fukuda | 25 | 13 | 16.1 | 65 | 33 | 13.7 | 21 | 0.394 | 1.274 | 0.754 | 1.816 | 0.02 | 0.267041 | 1.649273 | 3.744739 | 4.769495 | 0.935758 | 1.649273 |
| 35 | NK Annexin | (84) Brenu 2012 | EDTA | Fukuda | 25 | 14 | 16.1 | 65 | 32 | 13.7 | 21 | 0.438 | 1.146 | 0.632 | 1.681 | 0.02 | 0.263665 | 1.18211 | 3.792695 | 4.347517 | 0.419708 | 1.18211 |
| 36 | NK Annexin | (84) Brenu 2012 | EDTA | Fukuda | 25 | 4 | 32.2 | 65 | 28 | 13.7 | 21 | 0.143 | 0.823 | 0.321 | 1.341 | 0.02 | 0.256555 | -0.05911 | 3.897803 | 3.208982 | -0.13632 | -0.05911 |
| 37 | NK Annexin | (85) Marshall-Gradisnik 2016 | EDTA | Fukuda | 25 | 17 | 4 | 39 | 32 | 6 | 30 | 0.531 | 2.987 | 2.323 | 3.719 | 0.018 | 0.349718 | 6.184866 | 2.859448 | 8.540739 | 2.255589 | 6.184866 |
| 38 | NK Annexin | (86) Nguyen 2017 | EDTA | Intl | 1 | 3.4 | 2.6 | 15 | 8.9 | 4.6 | 25 | 0.387 | 1.354 | 0.664 | 2.098 | 0.018 | 0.354084 | 1.465514 | 2.824185 | 3.823414 | 0.683999 | 1.465514 |
| 39 | NK Annexin | (87) Eaton 2018 | EDTA | Fukuda | 6.25 | 2.3 | 8.5 | 8 | 2.2 | 2.4 | 9 | 1.045 | -0.016 | -0.999 | 0.966 | 0.015 | 0.461143 | -1.8588 | 2.168527 | -0.03393 | -1.00842 | -1.8588 |
| 40 | NK Annexin | (87) Eaton 2018 | EDTA | Fukuda | 12.5 | 2.3 | 4.2 | 8 | 5.5 | 4.5 | 9 | 0.418 | 0.696 | -0.282 | 1.749 | 0.015 | 0.476337 | -0.29949 | 2.099352 | 1.461379 | -0.22847 | -0.29949 |
| 41 | NK Annexin | (88) Balinas 2019 | EDTA | Fukuda | 25 | 6 | 6 | 10 | 16 | 13 | 10 | 0.375 | 0.946 | 0.035 | 1.941 | 0.015 | 0.453636 | 0.238218 | 2.204412 | 2.085144 | 0.045316 | 0.238218 |
| 42 | NK Annexin | (89) DuPreez 2021 | EDTA | Fukuda | 6.25 | 2 | 4.1 | 17 | 6 | 4.1 | 17 | 0.333 | 0.953 | 0.254 | 1.697 | 0.018 | 0.354246 | 0.32469 | 2.822896 | 2.688903 | 0.090725 | 0.32469 |
| 43 | NK Annexin | (89) DuPreez 2021 | EDTA | Fukuda | 12.5 | 6 | 8.2 | 17 | 10 | 8.2 | 17 | 0.6 | 0.476 | -0.204 | 1.18 | 0.018 | 0.339828 | -1.07277 | 2.942664 | 1.401493 | -0.74242 | -1.07277 |
| 44 | NK Annexin | (89) DuPreez 2021 | EDTA | Fukuda | 25 | 16 | 16.5 | 17 | 28 | 16.5 | 17 | 0.571 | 0.71 | 0.023 | 1.432 | 0.018 | 0.345778 | -0.37326 | 2.892033 | 2.053547 | -0.27524 | -0.37326 |
| 45 | NK Annexin | (90) Eaton-Fitch 2021 | EDTA | Fukuda | 6.25 | 4 | 1.9 | 15 | 7 | 5.8 | 15 | 0.571 | 0.676 | -0.054 | 1.445 | 0.018 | 0.365837 | -0.44534 | 2.73346 | 1.848708 | -0.37072 | -0.44534 |
| 46 | NK Annexin | (90) Eaton-Fitch 2021 | EDTA | Fukuda | 12.5 | 9 | 3.9 | 15 | 13 | 7.7 | 15 | 0.692 | 0.638 | -0.092 | 1.402 | 0.018 | 0.364677 | -0.55349 | 2.742151 | 1.748521 | -0.41971 | -0.55349 |
| 47 | PBMC 51Cr LU | (72) Barker 1994 | Heparin | Holmes | 17.7 | 38.8 | 26.7 | 16 | 119.75 | 115.4 | 12 | 0.324 | 1.011 | 0.23 | 1.852 | 0.017 | 0.394584 | 0.439768 | 2.534317 | 2.561886 | 0.136323 | 0.439768 |
| 48 | PBMC 51Cr LU | (73) Ojo-Amaize 1994 | Heparin | Holmes | 17.7 | 38 | 28.3 | 20 | 70 | 55.3 | 50 | 0.543 | 0.642 | 0.116 | 1.183 | 0.02 | 0.267216 | -0.74177 | 3.742284 | 2.40369 | -0.57356 | -0.74177 |
| 49 | PBMC 51Cr LU | (74) See 1996 IFNa | Heparin | Holmes | 10 | 89.1 | 18.9 | 15 | 125.7 | 24.7 | 20 | 0.709 | 1.595 | 0.85 | 2.414 | 0.017 | 0.384333 | 1.979332 | 2.601912 | 4.149525 | 1.086838 | 1.979332 |
| 50 | PBMC 51Cr LU | (74) See 1996 IFNa | Heparin | Holmes | 10 | 87.8 | 19.6 | 26 | 125.7 | 24.7 | 20 | 0.698 | 1.697 | 1.038 | 2.415 | 0.018 | 0.341702 | 2.531238 | 2.926528 | 4.966808 | 1.172581 | 2.531238 |
| 51 | PBMC 51Cr LU | (75) See 1997 Echin | Heparin | Holmes | 14.1 | 41.4 | 80.9 | 20 | 112.7 | 89 | 20 | 0.367 | 0.822 | 0.184 | 1.493 | 0.019 | 0.323261 | -0.05224 | 3.093478 | 2.541302 | -0.09073 | -0.05224 |
| 52 | PBMC 51Cr LU | (76) See 1998 Glyco | Heparin | Holmes | 14.1 | 42.3 | 141.2 | 91 | 114.7 | 151.2 | 30 | 0.369 | 0.501 | 0.085 | 0.923 | 0.022 | 0.21166 | -1.62439 | 4.724553 | 2.365631 | -0.93576 | -1.62439 |
| 53 | PBMC 51Cr LU | (77) See 1998 Homeo | Heparin | Holmes | 12.6 | 47.3 | 72.4 | 20 | 103.7 | 98.8 | 20 | 0.456 | 0.638 | 0.007 | 1.295 | 0.019 | 0.318039 | -0.63497 | 3.14427 | 2.005845 | -0.52094 | -0.63497 |
| 54 | PBMC 51Cr LU | (78) Levine 1998 | Heparin | Holmes | 17.7 | 19.9 | 5.1 | 8 | 83.6 | 50.4 | 8 | 0.238 | 1.681 | 0.581 | 2.976 | 0.013 | 0.558301 | 1.513468 | 1.791147 | 3.010974 | 0.803498 | 1.513468 |
| 55 | PBMC 51Cr LU | (91) Whiteside 1998 | Heparin | Fukuda | 17.7 | 54 | 30.4 | 8 | 123.6 | 133.3 | 51 | 0.437 | 0.548 | -0.203 | 1.314 | 0.017 | 0.378621 | -0.77067 | 2.641161 | 1.447668 | -0.684 | -0.77067 |

End SOM Table S2.

SOM Table S3. Random effects models for Hedges' g. Combined effect sizes were calculated separately for each cell source and method (columns) using Meta Essentials software (35–37)

| Combined Effect Size | All Data | WB 51Cr | PBMC 51Cr | PBMC Annexin | NK Annexin |
| --- | --- | --- | --- | --- | --- |
| Hedges' g | 0.962451 | 1.598763 | 0.606856 | 0.695248 | 1.076146 |
| Standard error | 0.106427 | 0.24467 | 0.166695 | 0.128452 | 0.209373 |
| CI Lower limit | 0.749079 | 1.060248 | 0.251555 | 0.412528 | 0.627085 |
| CI Upper limit | 1.175824 | 2.137278 | 0.962158 | 0.977968 | 1.525207 |
| PI Lower limit | -0.26607 | 0.116062 | -0.62377 | -0.00035 | -0.28167 |
| PI Upper limit | 2.190973 | 3.081464 | 1.83748 | 1.390841 | 2.433966 |
|  |  |  |  |  |  |
| Z-value | 9.043324 | 6.534361 | 3.640523 | 5.412523 | 5.139846 |
| One-tailed p-value | 0 | 3.19E-11 | 0.000136 | 3.11E-08 | 1.37E-07 |
| Two-tailed p-value | 0 | 6.39E-11 | 0.000272 | 6.21E-08 | 2.75E-07 |
|  |  |  |  |  |  |
| Number of subjects | 2982 | 899 | 915 | 495 | 673 |
| Number of data points | 55 | 12 | 16 | 12 | 15 |
|  |  |  |  |  |  |
| Heterogeneity | |  |  |  |  |
| Q | 270.4474 | 60.43276 | 69.11278 | 19.94756 | 57.36687 |
| pQ | 1.47E-30 | 7.7E-09 | 6.42E-09 | 0.046065 | 3.38E-07 |
| I2 | 0.800331 | 0.81798 | 0.782963 | 0.448554 | 0.755957 |
| T2 | 0.364156 | 0.393946 | 0.305563 | 0.08338 | 0.356952 |
| T | 0.603453 | 0.627651 | 0.552777 | 0.288755 | 0.597455 |

.

SOM Table S4. Egger Regression and Begg & Mazumdar Rank Correlation

| Egger Regression | Estimate | Standard error | CI LL | CI UL |  | Begg & Mazumdar |  |
| --- | --- | --- | --- | --- | --- | --- | --- |
| Intercept | 2.18 | 0.86 | 0.45 | 3.91 |  | ∆_x-y_ | 459.00 |
| Slope | 0.22 | 0.26 | -0.29 | 0.74 |  | Kendall's Tau a | 0.31 |
| t test | 2.53 |  |  |  |  | z | 3.33 |
| p-value | 0.014 |  |  |  |  | p | 0.001 |

.

SOM Table S5. Combined effect size estimates.

| Combined effect size | Observed |  | | Heterogeneity | |
| --- | --- | --- | --- | --- | --- |
| Hedges' g | 0.838258 |  | Q | | 339.9324 |
| Standard error | 0.040241 |  | pQ | | 4.13E-41 |
| CI Lower limit | 0.75758 |  | I2 | | 0.826436 |
| CI Upper limit | 0.918936 |  | T2 | | 0.453323 |
| PI Lower limit | -0.37428 |  | T | | 0.673292 |
| PI Upper limit | 2.050795 |  |  | |  |
|  |  |  |  | |  |
| Combined effect size | Adjusted |  | Trim and Fill | | On |
| Hedges' g | 0.749813 |  | Estimator for missing studies | | Leftmost Run/Rightmost run |
| Standard error | 0.039461 |  | Search from mean | | Left |
| CI Lower limit | 0.670852 |  | Number of missing studies | | 5 |
| CI Upper limit | 0.828773 |  |  | |  |
| PI Lower limit | -0.59975 |  |  | |  |
| PI Upper limit | 2.099379 |  |  | |  |

SOM Table S6. Failsafe tests. Meta-essentials (35–37).

| Rosenthal | |
| --- | --- |
| Overall Z-score | 21.44 |
| Failsafe-N | 9289 |
| Ad-hoc rule | FALSE |
| Gleser & Olkin | |
| Number of unpublished studies | 53 |
| Orwin | |
| Criterion value dc | 0 |
| Mean fail safe studies | 0.1 |
| Failsafe-N | 0 |
| Fisher | |
| Failsafe-N | 3571 |
| Chi-square test | 0.00E+00 |

SOM Table S7. Receiver operating characteristics (ROC) for cytotoxicity. The cytotoxicity of ME/CFS vs HC at each E:T ratio was compared for each cell source, method, combinations of cells and methods, LU and all data. Cytotoxicity at and below each threshold represents deficient NK function in ME. Optimal AUC, sensitivity and specificity were found at 25:1. The WB 51Cr method had the best separation between ME/CFS and HC. The threshold for published LU was 85.7 but the threshold was 15.7 when extrapolated to 20% cytotoxicity of HC. The PBMC with 51Cr studies of Caliguiri (9) and Mawle (71) were binned to 25:1 and 50:1.Results were provided for each combination of NK and PBMC with 51Cr and Annexin.

| All data combined | | | | | | | |
| --- | --- | --- | --- | --- | --- | --- | --- |
| Data | 6:1 | 12:1 | 25:1 | 50:1 | 1:1 extrapolated | All data |  |
| AUC | 0.673 | 0.841 | 0.905 | 0.875 | 1 | 0.704 |  |
| Significance | 0.26 | 0 | 0 | 0 | 0 | 0 |  |
| Threshold | 5.0 | 13.8 | 19.3 | 31.0 | 23.0 | 17.1 |  |
| Sensitivity | 0.714 | 0.765 | 0.846 | 0.667 | 1 | 0.764 |  |
| Specificity | 0.714 | 0.765 | 0.846 | 0.667 | 1 | 0.764 |  |
| Youden | 0.286 | 0.529 | 0.692 | 0.333 | 1 | 0.509 |  |
|  |  |  |  |  |  |  |  |
| Whole blood 51Cr method | | | | | | | |
| Data | 1:1 | 10:1 | 20:1 |  | All WB 51Cr |  |  |
| AUC | 1 | 1 | 1 |  | 1 |  |  |
| Significance | 0 | 0 | 0 |  | 0 |  |  |
| Threshold | 23 | 18.05 | 25.1 |  | 21.75 |  |  |
| Sensitivity | 1 | 1 | 1 |  | 1 |  |  |
| Specificity | 1 | 1 | 1 |  | 1 |  |  |
| Youden | 1 | 1 | 1 |  | 1 |  |  |
|  |  | | | |  |  |  |
| 51Cr with whole blood and PBMCs | | | | | | | |
| Data | 1:1 | 10:1 | 20:1 | 50:1 | LU | All 51Cr |  |
| AUC | 0.8 | 0.958678 | 0.938272 | 0.777778 | 0.951 | 0.843112 |  |
| Significance | 0.093533 | 0 | 4.96E-12 | 0.19836 | 0 | 2.49E-09 |  |
| Threshold | 19.5 | 16.97135 | 19.35 | 34.75 | 85.7 (15.7*) | 19.88304 |  |
| Sensitivity | 0.8 | 0.909091 | 0.888889 | 0.666667 | 0.778 | 0.821429 |  |
| Specificity | 0.8 | 0.909091 | 1 | 0.666667 | 0.778 | 0.928571 |  |
| Youden | 0.6 | 0.818182 | 0.888889 | 0.333333 | 0.556 | 0.75 |  |
| If cytotoxicity for ME/CFS values are extrapolated to match 20% cytotoxicity for HC, then the threshold is 15.7. | | | | | | | |
|  | | | | | | | |
| PBMC with 51Cr | | | | | | | |
| Test | 6:1 | 12:1 | 25:1 | 50:1 | All PBMC 51Cr |  |  |
| AUC |  |  | 0.828402 | 0.777778 | 0.726563 |  |  |
| Significance |  |  | 0.00059 | 0.19836 | 0.021973 |  |  |

**SOM FIGURES**

SOM Figure S1. Heterogeneity. The distribution of Hedges’ g values was .assessed in three ways. The standardized residuals were normally distributed but with significant outliers at both ends of the curve (A). The Galbraith plot reiterated these outliers (B). The quantile plot was linear (y = 2.258x + 0.242; R² = 0.969) suggesting the studies come from one population (C).

| 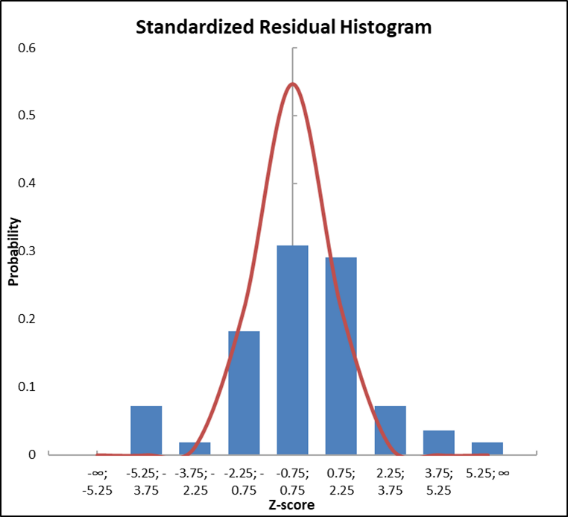 | 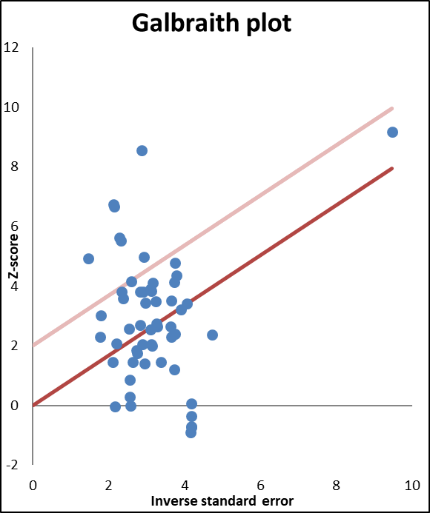 | 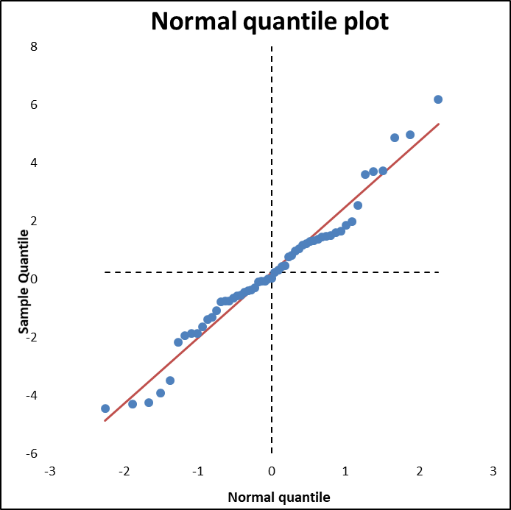 |
| --- | --- | --- |

.

SOM Figure S2. Cytotoxicity for ME/CFS versus HC for each data point. Regression lines of % cytotoxicity at each E:T ratio and study outcome were linear using published values for LU (A) as well as LU for ME/CFS extrapolated to 20% cytotoxicity in HC (B).

| 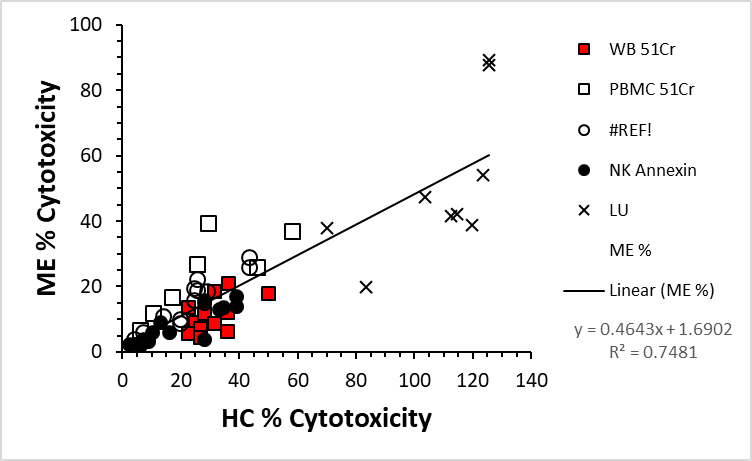 | 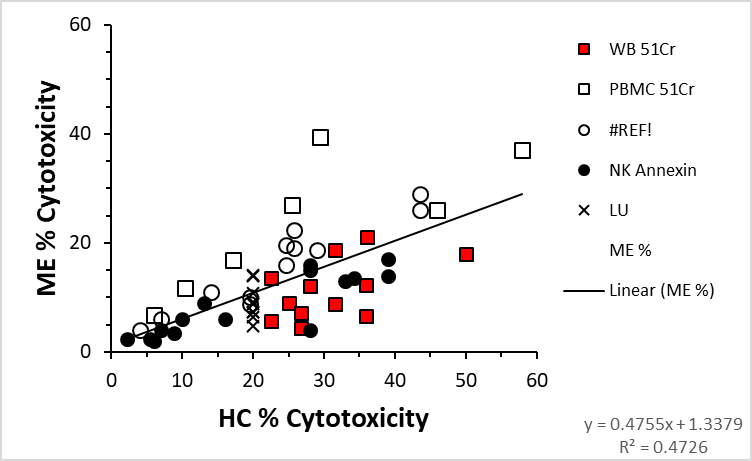 |
| --- | --- |

SOM Figure S3. ME/HC versus E:T. The ME/HC ratio for cytotoxicity was plotted for every E:T ratio. The average for all data was 0.569, SD=0.259 (n=55). The mean plus one SD was 0.828.

| 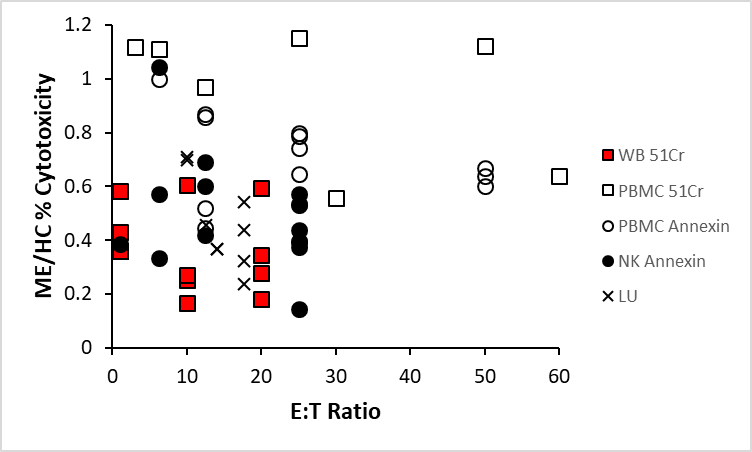 |
| --- |

Effects sizes were plotted for each E:T ratio (SOM Figure S4). Whole blood ^51^Cr gave the best results (g ≥ 0.97). The average was 1.02 (SD 0.83) [0.80 to 1.24] with mean + 1 SD = 1.86 and mean – SD = 0.19. High outliers (Figure 2) were the four data points from Masuda 2002 whole blood ^51^Cr study(70) and two cytometry points at 25:1 (85,93).. Low outliers were the PBMC ^51^Cr dose response with five E:T ratios of Mawle et al (71) with g = -0.13 and two cytometry studies [86,94). The outliers were no excluded from the final calculation of Hedges’ g.

| 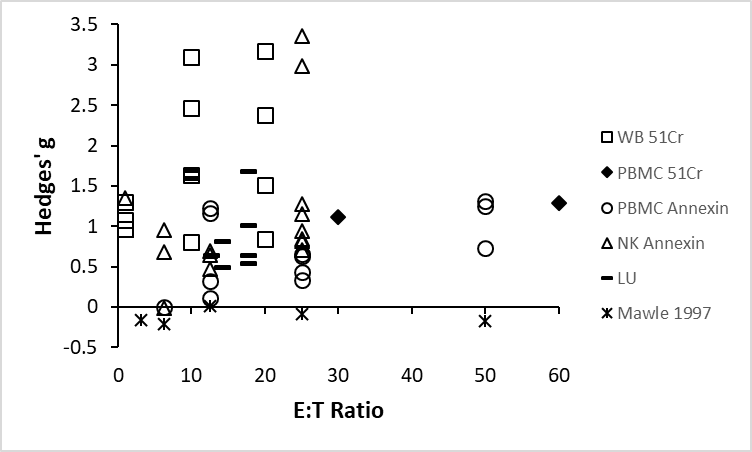 |
| --- |

SOM Figure S4. Hedges’ g for as a function of Effector : Target cell (E:T) ratio. Whole blood ^51^Cr (squares), PBMC 51Cr (black diamonds), PBMC annexin (open circles), NK annexin (open triangles), LU values (horizontal bars) were plotted versus E:T ratios. The PBMC ^51^Cr study of Mawle et al (71) (x) had Hedges’ g < 0 predicting a negative study.
